# The Impact of Shape-Based Cue Discriminability on Attentional Performance

**DOI:** 10.3390/vision5020018

**Published:** 2021-04-15

**Authors:** Olga Lukashova-Sanz, Siegfried Wahl, Thomas S. A. Wallis, Katharina Rifai

**Affiliations:** 1 Institute for Ophthalmic Research, University of Tübingen, 72076 Tübingen, Germany; siegfried.wahl@uni-tuebingen.de (S.W.); katharina.rifai@zeiss.com (K.R.); 2 Carl Zeiss Vision International GmbH, 73430 Aalen, Germany; 3 Amazon Development Centre Germany GmbH, 72076 Tübingen, Germany; thomas.wallis@tu-darmstadt.de

**Keywords:** endogenous attention, cue properties, cue processing

## Abstract

With rapidly developing technology, visual cues became a powerful tool for deliberate guiding of attention and affecting human performance. Using cues to manipulate attention introduces a trade-off between increased performance in cued, and decreased in not cued, locations. For higher efficacy of visual cues designed to purposely direct user’s attention, it is important to know how manipulation of cue properties affects attention. In this verification study, we addressed how varying cue complexity impacts the allocation of spatial endogenous covert attention in space and time. To gradually vary cue complexity, the discriminability of the cue was systematically modulated using a shape-based design. Performance was compared in attended and unattended locations in an orientation-discrimination task. We evaluated additional temporal costs due to processing of a more complex cue by comparing performance at two different inter-stimulus intervals. From preliminary data, attention scaled with cue discriminability, even for supra-threshold cue discriminability. Furthermore, individual cue processing times partly impacted performance for the most complex, but not simpler cues. We conclude that, first, cue complexity expressed by discriminability modulates endogenous covert attention at supra-threshold cue discriminability levels, with increasing benefits and decreasing costs; second, it is important to consider the temporal processing costs of complex visual cues.

## 1. Introduction

On a regular day, our attention is guided by multiple visual cues, whether we are aware of them or not. In many contexts, purposely designed visual cues can influence human behavior through attentional guidance. To name a few, it can be when browsing on a web page of an online shop with a user-friendly design or driving from work to home using navigational assistance. With rapidly developing technology, visual cues become a powerful tool to deliberately guide human’s attention, (e.g., [1,2]). A systematic design of visual cue properties is then necessary for intended guidance. Thus, it is of importance to know how manipulation of cue properties affects attention. From extensive research, it is well known that when we give our attention to one location, our visual performance generally enhances in that region, however it decreases in other non-attended locations due to limited processing capacity [3,4,5,6,7,8,9,10]. The attentional costs in the unattended locations become essentially a bottleneck for the design of visual cues aiming to deliberately improve human’s performance [11,12]. Considering applications such as signage design or augmented reality, a potential benefit of rather subtle visual aids is emerging in contrast to strong conventional symbolic cue stimuli, to improve performance where it is intended to be improved, but not significantly decrease the performance in the remaining tasks [13]. A gradual manipulation of cue impact would allow a very controlled attentional guidance through visual spatial cueing. Previously, visual cues have been manipulated parametrically, where some studies reported gradual change in visual performance when varying cue properties such as its size or contrast (e.g., [14,15]), whereas others argued for lack of flexibility of attentional allocation (e.g., [16]). In the present verification study, we intended to systematically modulate attentional benefits and costs via the spatial design of the cue, namely, its shape. However, increasing the cue design complexity, aiming to improve the benefits and costs relation, generally might increase the time to process the cue. In the present study, we examine if there are additional temporal costs in performance due to the processing of more complex cues.

One way to facilitate an attentional shift in a classical attentional paradigm is to precue a target location with a spatial cue preceding the target stimulus onset [17]. Numerous studies investigated the modulation of attention deployment when varying the target, distractors, or the task in an array of attentional paradigms (see [18,19]). Several studies have examined the modulation of the properties of the cue itself (e.g., [15,20,21,22]). Regarding a gradual modulation of visual cue properties, previously it was shown that attentional benefits and costs scaled with the contrast of an exogenous spatial cue, indicating a gradual prioritizing of attentional resources allocation for the exogenous attention [15]. In many circumstances, our attention is guided by endogenous visual cues, which in turn, can vary in complexity [23,24,25].

In the present verification study, we address how modulation of cue complexity affects attentional performance. In the context of this work, the cue discriminability was used as a proxy of the cue complexity. The more difficult it is to discriminate the cueing direction, the more complex the cue becomes. It is investigated how the manipulation of cue discriminability of an endogenous spatial cue, even above discrimination threshold, modulates endogenous covert attention. Specifically, in an orientation-discrimination two-alternative-forced choice (2AFC) task, it is studied how the discriminability of an endogenous cue impacts attentional performance in the cued and not cued locations. A shape-based spatial cue was designed where through a systematic manipulation of the shape of the cue, a set of cues of various cue discriminability levels was generated, including supra-threshold levels of cue discriminability. Performance in the valid, invalid, and neutral conditions was assessed. By doing so, the benefits and costs of the allocated attention were quantified. We hypothesize that if the cue discriminability of an endogenous cue is increased, the benefits and costs of the cue will also grow in magnitude across the cue discriminability even when the cued direction is fully discriminated.

Less discriminable cues, even at discriminability level over the sensitivity threshold, generally might require more time to process. This can influence the time course of an attentional effect [23]. From an extensive body of literature, it is known that endogenous attention has a specific time span approaching its maximum at ∼300 ms after the cue onset (e.g., [26,27,28,29,30]). In the present verification study, we intended to evaluate temporal delays of attentional shift through cue processing. Specifically, to decode a less discriminable cue, above the discrimination threshold, one would need a longer time; thus, attentional performance can benefit from a longer period between the cue offset and stimuli onset, i.e., inter-stimulus interval. The inter-stimulus interval is linked to another common temporal measure in attentional paradigms, stimulus-onset asynchrony (SOA), where the latter is the time between the cue and stimulus onsets. In the present study, to address the temporal effect of the cue discriminability on attentional performance, conditions with different inter-stimulus intervals were compared.

## 2. Methods

### 2.1. Participants

In this verification study, 11 naive participants with an average age of 24.4 ± 3.7 years were tested. Eight participants were female, three participants were male. All procedures conformed to Standard 8 of the American Psychological Association’s “Ethical Principles of Psychologists and Code of Conduct (2010)”. The study was approved by the ethics committee of the Faculty of Medicine of the University of Tübingen. Signed informed consent was obtained from each participant prior to the measurements.

### 2.2. Apparatus and Stimuli

#### 2.2.1. Apparatus

The visual stimuli were presented on a VIEWPixx monitor (VPixx Technologies Inc., Montreal, QC, Canada) with a refresh rate of 60 Hz, resolution of 1920×1080 pixels, at a distance of 50 cm from the monitor. The paradigm was generated using the PsychoPy library [31] and an additional Python package for integrating eye-tracking, PyGaze [32], running on a Windows 10 PC. All experiments were performed in a dark room. To ensure covert attentional shift and exclude possibility of saccades towards the target stimuli, all participants were instructed to maintain their fixation at the central location at all times throughout the experiment. The fixation was monitored using an EyeLink 1000 Plus (SR Research, Ottawa, ON, Canada) eye tracker. In case the participant looked away from a defined area of 2.5${}^{\circ }$ of diameter during the stimuli display, the respective trial was withdrawn and a new trial started. The head was stabilized with a chin rest. Responses of participants were registered with an external keyboard.

#### 2.2.2. Shape-Based Spatial Cue Generating

An intuitive choice of an endogenous cue is an arrow pointing to a certain direction. Overlearning of this stimulus, however, limits the possibility to gradually modulate the cue property (e.g., [23,33]). To avoid a binary attention allocation, we propose a novel cue design where a parametric shape manipulation enables progressive modulation of cue discriminability.

**Figure 1:**
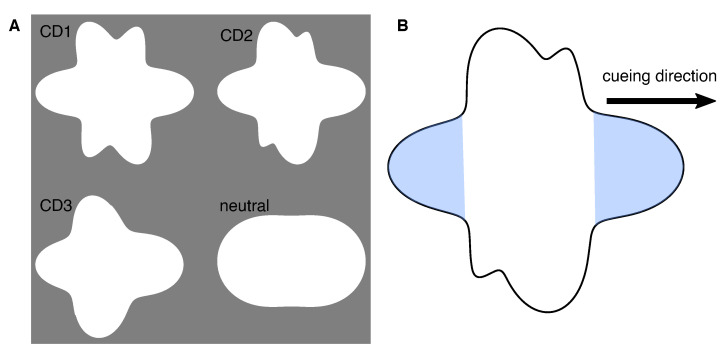
The shape-based cue construction. (**A**) At different cue discriminability levels: low (CD1), medium (CD2), and high (CD3), together with the neutral cue. (**B**) Schematic representation of cue direction identification. The “largest” lobe of the left and right lobes, estimated by the area of the lobe, indicates cueing direction. In this figure, as an example, all presented shape-based cues indicate the direction to the right. In the experiment, the right and the left directions were cued equally often.

As an endogenous shape-based direction-indicating cue a filled irregular radial frequency pattern was used. Sinusoidal modulation of the radius *R* at polar angle θ in a radial frequency pattern is generally described by Equation (1) [34]:

(1) $$R\left(\theta \right)=R_{0}\left(1+A\;\cdot \;\sin\left(\omega \theta +\phi \right)\right),$$

where $R_{0}$ is the mean radius, *A* is the radial modulation amplitude, ω is the radial frequency, and ϕ is the angular phase of the pattern. In an irregular radial frequency pattern, the radial frequency varies between different lobes. In this study, filled irregular radial frequency patterns with six cycles of sinusoidal modulation were used. For details on generating the cue please refer to the Supplementary Information Section S1 and Figure S1. To indicate cueing direction, the radial frequency of one of the lobes of the pattern was modulated in order to make it larger compared to the rest of the five lobes. In particular, to identify the cued direction, participants were to compare areas covered by the right and left lobes: the largest of two indicated the cueing direction, left or right. The ratio between the modulation factors for the radial frequency of two relevant lobes determined cue discriminability level: the smaller the radial frequency at a given amplitude and mean radius of the cueing lobe is, the larger the cueing lobe compared to the opposite lobe is; thus, the more discriminable is the cue. When systematically modulating the radial frequency of one lobe, the radial frequency of the opposite lobe was kept constant and the remaining four lobes were adjusted such that the total filled area of the cue stayed unchanged. The ratio of the modulation factor of the cueing lobe and the opposite lobe was systematically varied from 0.98 down to 0.68 with a step of 0.02, corresponding to the least and the most discriminable cues, respectively. The angular phase was adjusted for each cue such that the cueing and the opposite lobes are oriented horizontally. As a neutral cue, a two-lobe regular radial frequency pattern was used, the lobe amplitude of which was adjusted to keep the filled area equal to that of the irregular patterns. In Figure 1, examples of cues used in the experiments are demonstrated. Essentially, the arbitrary units of cue discriminability are determined by the modulating factor of the radial frequency for the cueing lobe. The values of 0.96, 0.84, and 0.68 were selected as the modulating factor ratios for the cues used in the main experiment. These values were selected based on a cue sensitivity test conducted before the main experiment (see details in Section 2.3.2 and Section 2.4.1). In the context of this study, the discriminability levels of these cues are, respectively, denoted by CD1, CD2, and CD3, in arbitrary units.

The type of the cue is defined to be endogenous following the definitions of voluntary attentional shift induced by a central cue (see [3]). Furthermore, to some extent the designed shape-based cue shares some cueing properties with a conventional arrow cue such as pointing towards a certain direction. Although previously it was demonstrated that arrows induce a fast attentional shift which can be debated as automatic stimulus-driven, the neurophysiological evidence suggests that this fast shift is due to an overlearned association mechanism rather than exogenous attentional process (e.g., [23,33]). Due to a novel cue design in the present study, overlearning is not to be expected. Thus, the cue in the present study is considered to be endogenous.

#### 2.2.3. Stimuli

As stimuli, two tilted Gabor patches were used of spatial frequency 2 cpd, visual angle 2${}^{\circ }$, and Michelson contrast 50% located at 4${}^{\circ }$ eccentricity left and right from the central fixation cross. The magnitude of the tilt angle of the Gabor patches tested in the experiment was fixed to a set of five different values: 0.5${}^{\circ }$, 1${}^{\circ }$, 3${}^{\circ }$, 5${}^{\circ }$, and 12${}^{\circ }$ relative to the vertical. The magnitude of the tilt angle and the tilt direction of two stimuli simultaneously presented in each trial were independent of each other.

### 2.3. Experimental Procedure

#### 2.3.1. General Procedure

The study consisted of three sessions, each performed on different days. The first session consisted of three experiments: a cue sensitivity test with the shape-based cue, a reference cue sensitivity test with a line cue, and a practice session for the main experiment. During the second and the third sessions, on two different days, participants performed the main experiment with two different inter-stimulus intervals, respectively. The inter-stimulus interval was fixed for a given experimental session. Each experimental session lasted approximately one hour, where participants performed the experimental task for a maximum of 15 min in a row. In particular, the second and third sessions were split into three sub-15 min sessions with a short break in between to let the participants rest. During breaks, participants remained in the same room under unchanged lighting conditions. For each participant, the number of days between the experimental sessions varied from three to seven days.

#### 2.3.2. Cue Sensitivity Test

Before the beginning of the first session, cues of various cue discriminability were demonstrated and explained to the participants. The participants were allowed to familiarize themselves with the cues. In the cue sensitivity test, participants were presented with a spatial cue for 150 ms indicating one of the two locations, left or right of the central fixation cross. After the cue display, participants were instructed to report the perceived cue direction with a keypress as fast as possible upon the cue offset. Therewith, the response times for different cues for each participant were registered. When a neutral cue was presented, participants were asked to press either of the two response keys. The neutral cue differed from the informative cues by its symmetric design to avoid its confusion with a very subtle informative cue. In the cue sensitivity test with shape-based spatial cues, participants were presented with cues of 16 different cue discriminability levels. Each cue discriminability level was presented 20 times randomly alternating between different cue discriminability levels and the neutral cue, as well as in random order of left and right cueing directions. In addition to the cue sensitivity test with a shape-based cue, each participant performed a separate line cue sensitivity test, where the spatial cue was a black line of size 0.7${}^{\circ }$ and the neutral cue was a black dot of radius 0.2${}^{\circ }$. The line cue spatially cued the participant either towards the left or the right location, depending on which side from the center it appeared. The line cue test was performed directly after the shape-based cue sensitivity test with a short 3–5 min break. During the break, the participant remained in the same room and same lighting conditions.

In total, each participant performed (16 cue discriminability levels +1 neutral cue) × 20 trials resulting in 340 trials during the cue sensitivity test with shaped-based cue, and (1 line cue +1 neutral cue) × 20 trials resulting in 40 trials during the line cue sensitivity test.

#### 2.3.3. Practice Session

The purpose of the practice session was for the participants to familiarize themselves with the procedure of the main experiment. The data obtained during the practice session were not used for further analysis. The experimental flow of the practice session was identical to the main experiment, only, instead of varying target stimuli tilt angles, a fixed large angle of 20${}^{\circ }$ for training purpose was used. The practice session consisted of 48 trials.

#### 2.3.4. Main Experiment

**Figure 2:**
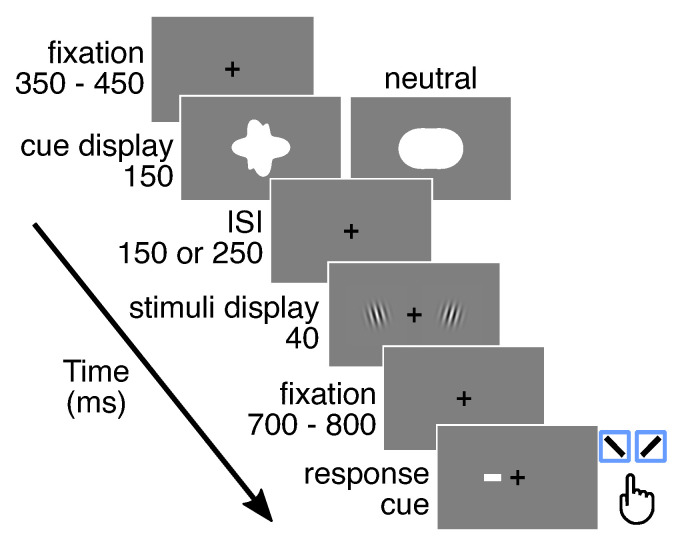
Experimental flow of the main experiment.

In the main experiment, participants performed a direction-discrimination 2-alternative-forced-choice (2AFC) task in a modified Posner attentional paradigm (Figure 2). After a variable period of fixation between 350 ms and 450 ms, a shape-based direction-indicating cue was presented for 150 ms. Thereafter, participants shifted their attention to the cued location while fixating in the center. Next, after an inter-stimulus interval, two stimuli were presented simultaneously for 40 ms: one on the left and one on the right side of the center (see the detailed description of the stimuli in Section 2.2.3). Directly after the offset of the stimuli, a white line of 1.5 degree visual angle (the response-cue) was presented at fixation position indicating the final target stimulus location. The approach to use a response-cue to collect the responses in attended and unattended locations was previously used (e.g., [7]). At the end of each trial, participants had to report the orientation of the target stimulus. The response cue disappeared upon the participant’s response. The participant reported the tilt orientation of the target, clockwise (CW) or counterclockwise (CCW), with a keypress right or left, respectively. Two different inter-stimulus intervals were used: 150 ms and 250 ms. In the context of this study, as the cue display duration was fixed, the two experimental conditions are defined by the inter-stimulus interval instead of stimulus onset asynchrony. The smaller ISI 150 ms was selected based on the time period ∼300 ms after the onset of the cue when endogenous attention typically approaches its maximum, as also described in the introduction (e.g., [26,27,28,29,30]). In the present study, the idea was to test how benefits and costs evolve with systematic cue modulation, but at the same time monitor the potential temporal processing disadvantage of the cues. Considering the novel design of the cue, in addition to the conventional ISI, a second longer ISI 250 ms was selected. The time interval between the stimuli offset and the response-cue onset was set randomly between 700 ms and 800 ms. To ensure that participants shifted attention to the cued location, the number of valid trials at a given cue discriminability level was set to be twice as large as the number of invalid trials. In addition, one-quarter of the total amount of trials at a given cue discriminability level was neutral trials. At a given cue discriminability level and target stimulus tilt angle, there were 6 measurement points collected for the invalid, 12 for the valid, and 6 for the neutral conditions. This limited number of measurements per data point was chosen due to lengthy and focus-demanding experimental sessions. The validity of the cue was, thus, 67%, which has been previously used (e.g., [35]). In each of two sessions during the main experiment, each participant performed 720 trials resulting in 1440 trials in total.

### 2.4. Analysis

#### 2.4.1. Cue Sensitivity Test

From the cue sensitivity test with a shape-based spatial cue for each participant, the proportion of correct responses as a function of cue discriminability was obtained. The data were fitted with the Weibull psychometric function with a chance level of 50%, resulting in a cue sensitivity curve for each participant. The proportion correct values at a given cue discriminability were averaged over all participants. The present study was aimed to test a possible scaling of attentional performance with cue discriminability. Given that the task was rather challenging for the participants, the possible duration of the experimental session was constrained by the fatigue of the participants. Therefore, a set of three cue discriminability values was selected for the main experiment as a minimum set size to evaluate the gradual effect. Furthermore, although the novel cue design enabled an incrementing cue sensitivity supported by the cue sensitivity test, it also had some constraints in terms of the maximum cue discriminability possible. For the main experiment, the cue with CD3—the maximum allowed by the cue design—was selected. The second value of cue discriminability CD2 was selected approaching the saturation point of the cue sensitivity curve. Both CD2 and CD3 correspond to a high cue sensitivity represented by proportion correct ∼95%; nonetheless, they differ based on the relative horizontal lobes size. Finally, the third value of CD1 was selected to complete a set of three cue discriminability levels. The value was chosen to be different from CD2 and CD3 but still to be significantly over the chance level with respect to proportion correct in the cue sensitivity test.

#### 2.4.2. Main Experiment

In the orientation-discrimination task for each participant, a ratio of CW responses to the total amount of responses was determined at a given target stimulus tilt angle. Value 1 of this ratio corresponds to all responses at a given target stimulus tilt angle being CW, and value 0—to all responses at a given target stimulus tilt angle being CCW. The CW tilt angles relative to the vertical are denoted with positive values, whereas the CCW tilt angles are denoted with negative values. Stimulus response curves were analyzed separately for a given inter-stimulus interval, cue discriminability, and validity. The proportion of CW responses as a function of the target stimulus tilt angle was fitted with a psychometric function fixing asymptotes to 0 and 1. The slope of the psychometric function at the point of subjective equality (PSE) was used as a measurement of performance in attended and unattended locations, corresponding to the valid and invalid conditions, respectively. To evaluate attentional benefits (valid) and costs (invalid) of the cues, the slopes in the valid and invalid conditions were normalized to the slope in the neutral condition and referred further in the manuscript as just slopes. An impact of cue discriminability on attentional performance was estimated from the difference in slope in the valid and invalid conditions. The main effects as well as the interaction between two factors—cue discriminability and inter-stimulus interval—were then statistically evaluated by performing a two-way repeated-measures analysis of variance (ANOVA) with valid–invalid slope difference as the dependent variable, followed by a post hoc analysis.

#### 2.4.3. Cue Processing Temporal Cost

To estimate an effect of cue processing on attentional performance, the difference in performance at two different inter-stimulus intervals was statistically evaluated within the repeated-measures ANOVA. Furthermore, the correlation between individual cue response times and performance in the valid/invalid conditions at two different inter-stimulus intervals was assessed. We assumed the total response time to be a sum of a “baseline” response time and a specific cue processing time. The “baseline” response time is considered here to be the response time to a line cue, a widely used simple endogenous visual cue [3]. Therefore, as an approximation of the specific shape-based cue processing time, the response time of a line cue for each participant was subtracted from the total response time of the corresponding shape-based cue. The resulting processing time was then correlated with the performance difference in the sessions with different inter-stimulus intervals. The performance difference was estimated by a difference in slope at ISI 250 ms and ISI 150 ms. A positive correlation between the reaction time of slope difference would denote that participants with longer cue processing performed better in the session with a longer inter-stimulus interval. Pearson correlations were computed for the three cue discriminability levels separately at a given validity.

## 3. Results

### 3.1. Cue Sensitivity

**Figure 3:**
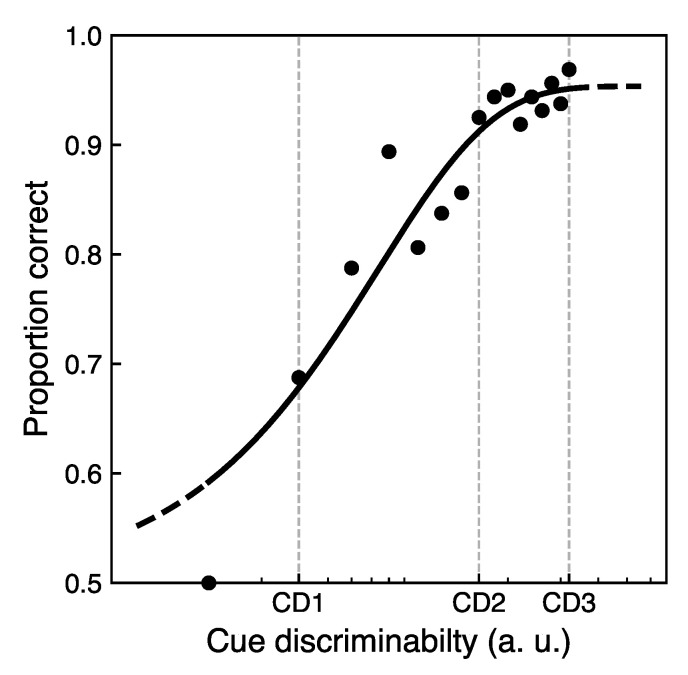
Results of cue sensitivity test with a shape-based cue: mean proportion correct averaged over all participants as a function of the cue discriminability. The grid lines correspond to three cue discriminability levels in arbitrary units selected for the main experiment.

Figure 3 shows the mean proportion correct values averaged over all participants as a function of cue discriminability level. The proportion correct increases with stimulus discriminability level. Based on these data three cue discriminability levels, CD1, CD2, and CD3 were selected. The individual response times as a function of the cue discriminability levels are shown in Figure 6 in Section 3.3, where it is more relevant.

### 3.2. Main Experiment

The individual fits for all measured participants can be found in Supplementary Information Figure S3–S24. Out of 11 participants, three participants were excluded (see Supplementary Information Figures S9 and S10 and Figures S21–S24). For two of the excluded participants, the task appeared to be too difficult, which was indicated by the feedback from participants as well as by consistently high noise level of the fitted psychometric functions for all experimental conditions. Another participant explicitly reported by himself that he did not follow instructions of the task as suggested, specifically, he did not use the cue. The data sets collected from 8 participants were used for the final analysis. The goodness of the fit can be described by deviance extracted from the fitting models (see Supplementary Information Figure S2). The distribution of the deviance was originally planned to be used as an objective exclusion criterion. It largely varies across the validity, which is expected as the invalid condition is particularly challenging. It also has fewer measured data points than valid or neutral conditions to keep the valid–invalid trials ratio sufficient for the cue to remain informative, which led to higher noise levels in the invalid condition. Given the difficulty of the experimental design, large number of fits per participant, and limitation in the duration of the experiment to avoid participants’ fatigue, it was rather challenging to strictly apply the objective exclusion criterion. For future studies, a larger sample size, as well as more measurement points per participant, could improve the goodness of the fits and enable following objective guidelines to exclude participants. For the purpose of this verification study to obtain preliminary data on the cue discriminability effect on attentional performance, the above-described data set was used, where a rather subjective exclusion criterion was applied. (It is worth mentioning that when the full participants set counting also excluded participants was tested, the statistical outcome did not change notably. In particular, there was a significant effect of cue discriminability between slopes in the valid and invalid conditions ($F\left(2,20\right)=8.53,\;p<0.01,\;\eta_{p}^{2}=0.46$). The effect of inter-stimulus interval was not significant ($F\left(1,10\right)=3.93,\;p=0.08,\;\eta_{p}^{2}=0.28$). The interaction between cue discriminability and ISI was not significant ($F\left(2,20\right)=1.48,\;p=0.25,\;\eta_{p}^{2}=0.13$)).

**Figure 4:**
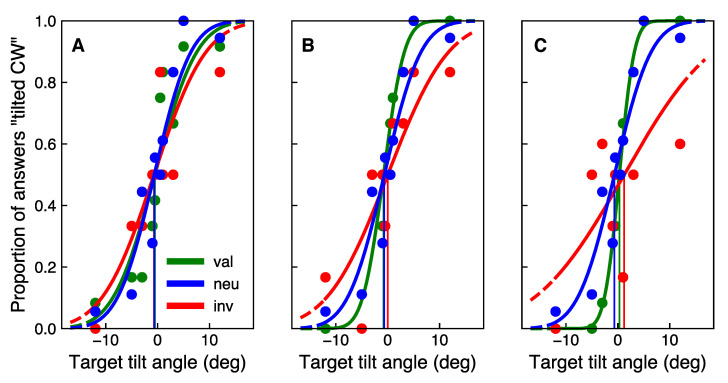
An example of a family of curves obtained from one session (ISI 150 ms) for one participant. The proportion of answers “tilted CW” as a function of the target stimulus tilt angle is fitted with a psychometric function. Green, blue, and red curves correspond to the valid, neutral, and invalid conditions, respectively. The data sets correspond to cue discriminability levels (**A**) CD1, (**B**) CD2, and (**C**) CD3. Note that data points for some tilt angles are superimposed—all tilt angles were equally shown to the participants.

Figure 4 shows response curves of one example participant, sorted in the valid, invalid, and neutral conditions. Three cue discriminability levels are shown in three subgraphs. Different colors of the curves correspond to different validity conditions. In this example, as well as for most of the participants, for the CD2 and CD3 cue discriminability levels, the invalid curve is more shallow compared to the valid and neutral curves, whereas the neutral curve lies in between the valid and invalid. Thus, slopes tend to be steeper in the valid condition, compared to the invalid condition. This trend becomes more pronounced with increasing cue discriminability level which serves as a hint that the cue discriminability affects the performance.

**Figure 5:**
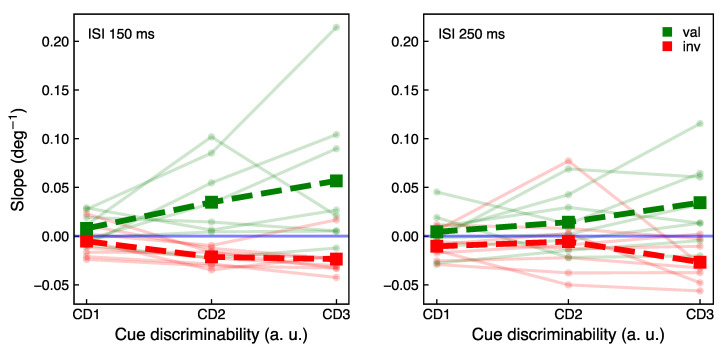
The average slope of response curves over all participants (dashed lines) for valid (green), invalid (red), and neutral (blue) conditions at different cue discriminability levels, together with single-participant data (continuous semi-transparent lines).

In Figure 5, the mean results overlaid with the individual data are depicted. The slopes were normalized by subtracting the slope in the neutral condition; thus, the neutral level depicted in Figure 5 is independent of the cue discriminability level and lies at zero. The group data with unnormalized data in the neutral, valid, and invalid conditions can be found in the Supplementary Information.

From the two-way repeated-measures ANOVA, first, we found a significant effect of cue discriminability on the difference between slopes in the valid and invalid conditions ($F\left(2,14\right)=7.62,\;p<0.01,\;\eta_{p}^{2}=0.52$). To evaluate which subgroups of measurements are different, we performed a post hoc analysis which revealed for ISI 150 ms a significant difference between the slope difference at cue discriminability levels CD1 and CD2. (p<0.05), and for ISI 250 ms a significant difference between slope difference at CD1 and CD3. (p<0.05). The difference between the slope difference at CD2 and CD3 levels for ISI 250 ms was close to but not significant (p=0.14). The effect of cue discriminability suggests that an easier more discriminable cue has a stronger attentional effect than a more complex less discriminable cue. This result suggests the impact of a shape-based cue discriminability on attentional performance.

The effect of inter-stimulus interval is not significant (F(1,7)=4.81, p=0.06, $\eta_{p}^{2}=0.41$). The interaction between two factors—the cue discriminability and inter-stimulus interval—is also not significant ($F\left(2,14\right)=1.01,\;p=0.39,\;\eta_{p}^{2}=0.13$). The absence of a significant difference in performance at different inter-stimulus intervals might originate from an individual variation in cue processing times obtained from the cue sensitivity test. The difference in performance at distinct inter-stimulus intervals might be influenced by individual variances between participants. In Section 3.3, the correlation of the individual performance at different inter-stimulus intervals with the cue processing time was evaluated.

The effect size of the cue discriminability represented by partial eta squared is large according to Cohen’s rule of thumb [36]. Note, however, that the large effect size is also influenced by a small sample size tested in the present study. Beyond the scope of this verification study, future work with extended number of participants is necessary to target more representative effect size.

### 3.3. Cue Processing Temporal Costs

**Figure 6:**
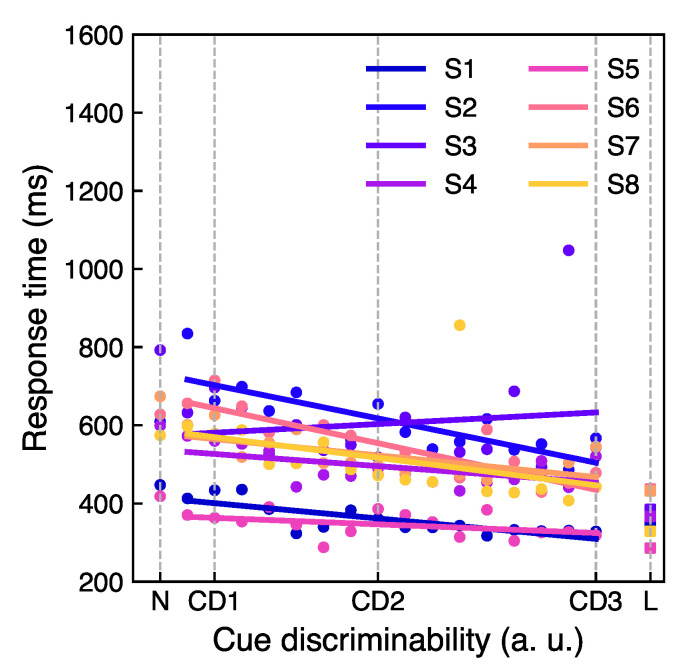
The individual response time recorded in the cue sensitivity test as a function of cue discriminability. The N tick label corresponds to the neutral cue. On the right, the line cue response times are depicted, denoted by a tick label L. The straight solid lines are the linear fits of the measurement points for each participant. Different colors correspond to different participants.

In Figure 6, individual response times as a function of the cue discriminability levels are shown fitted with a linear function. The response times for the neutral and the line cues for each participant are also depicted in Figure 6. As expected, the general trend for all the participants is a decreasing response time with increasing cue discriminability. The individual response times range from approximately 300 ms to 700 ms, and the line cue response times lie between 250 ms and 400 ms. By subtracting the line cue response time as a baseline from the response time at a given cue discriminability, the corresponding shape-based cue processing time was determined. Analyzing the wide range of cue processing times varying among participants, we suggest that some participants were “faster” and some were “slower” in cue processing. In particular, from the analysis of individual slopes at different inter-stimulus intervals, for some participants performance was better (the slope was larger) at ISI 250 ms than at ISI 150 ms at all tested cue discriminability levels, whereas for some other participants it was the opposite. This suggests that some participants benefited from a longer interstimulus interval, whereas the others did not. The higher reaction times measured for the neutral cue are suggested to be caused by a decision process, as the participant knew that the cue was not cueing any specific direction, but the response still had to be made.

**Figure 7:**
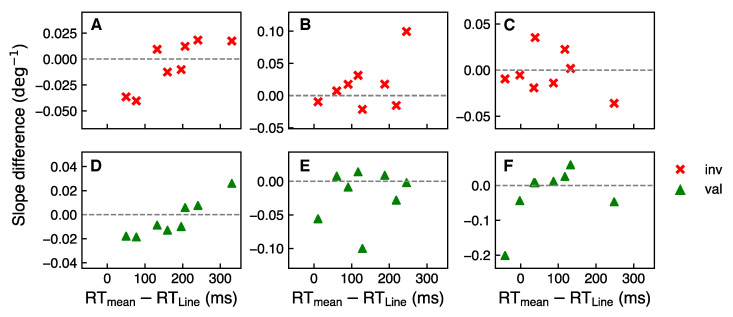
The difference between individual slopes at ISI 250 ms and ISI 150 ms as a function of the cue response time normalized to the line cue response time at a given cue discriminability and validity. Red crosses and green triangles represent invalid and valid conditions, respectively. (**A**,**D**) CD1 cue discriminability; (**B**,**E**) CD2; (**C**,**F**) CD3. A positive Pearson correlation for the valid and invalid conditions at CD1 was obtained with Pearson coefficients 0.83 and 0.93, respectively.

Figure 7 shows the difference between individual slopes at ISI 250 ms and ISI 150 ms as a function of cue processing time at a given cue discriminability and validity. Smaller abscissa values correspond to “faster” participants, that is, participants with shorter cue processing times and larger abscissa values correspond to “slower” participants, that is, with longer cue processing times. Negative values on the y-axis represent a larger slope, i.e., a better performance, at ISI 150 ms compared to ISI 250 ms at a given cue discriminability and validity condition for respective participants.

From the Pearson correlation test, it was found that there is a positive correlation for the least discriminable cue CD1 for both valid and invalid conditions with Pearson coefficients 0.93 (p<0.05) and 0.83 (p<0.001), respectively. Even though correlations based on a small sample can be noisy, this result suggests that for CD1 cue discriminability level the “slower” participants indeed benefit from additional 100 ms between cue offset and stimuli onset, compared to the “faster” participants with larger attentional effect at a shorter interstimulus interval.

## 4. Discussion and Conclusions

In a verification study, the impact of a shape-based endogenous cue discriminability on attentional performance was evaluated in a 2AFC orientation-discrimination task. Applying a gradual systematic shape-based design, a set of cues with different cue discriminability levels was generated, which were then tested in a cue sensitivity test (Figure 3). For the main experiment, three different levels of cue discriminability represented by the relative size of two horizontal lobes of the cue—CD1, CD2, and CD3—were selected. Attentional benefits and costs of the cue were evaluated comparing performance in the valid and invalid conditions at a given cue discriminability. Repeated-measures ANOVA results revealed a significant impact of cue discriminability on attentional performance. Furthermore, the temporal costs due to cue processing were addressed. In particular, the difference in the attentional performance at two tested inter-stimulus intervals positively correlated with the individual cue processing times for the low cue discriminability CD1.

Previously, some studies demonstrated a gradual change in visual performance when varying cue properties (e.g., [14,15]), whereas others argued for lack of flexibility of attentional allocation (e.g., [16]). Interestingly, Fuller et al. [15] showed that manipulation of an exogenous cue modulates appearance even at supra-threshold contrast levels of the cue, indicating the flexibility of attention. The results of the present verification study preliminarily suggest an increasing trend of the effect of cue properties modulation on endogenous covert attention. These findings support the hypothesis that through a careful systematic design of a visual cue, in this case, based on its shape, a gradual distribution of attention appears to be possible. This, in turn, gives an insight into potential visual cues aiming to subtly and deliberately guide person’s attention.

More complex cues, even at a discriminability level above the threshold, generally can take more time to process. In [23], in a detection task, authors found a stronger attentional effect at longer SOA when comparing eye-gaze and arrow cue with a more complex texture cue. In the present study, it was investigated whether additional processing time of less discriminable cues affects the temporal course of attentional effect. We addressed the hypothesis that modulation of cue design with a purpose to improve performance can come at a cost of additional cue processing. Based on performance differences at two different ISI and its correlation with individual cue processing times, it was found that “slower” participants who presumably needed more time to process a less discriminable cue performed better at a longer ISI, in contrast to “faster” participants who needed less time for cue processing. This result can be explained if to assume a subsequent attentional mechanism on a time scale where the participant, first, decodes the cue and thereafter shifts attention to the cued location approaching attentional maximum at approximately 300 ms after the cue onset. With this assumption, at a shorter inter-stimulus interval, attention is already close to its maximum for the “faster” participants. In contrast, for the “slower” participants at shorter interstimulus interval attention does not yet reach its maximum. At a longer interstimulus interval, however, the “faster” participants are already past the maximal attentional performance, whereas the “slower” participants approach the maximum. Considering the temporal range of the present experiment, these individual differences in the performance at different ISI could also be linked to the inhibition of return phenomena, when the attentional benefit in the cued location is observed only within a limited time period [37,38].

A possible question is, why a positive correlation between the attentional performance at two interstimulus intervals with the individual cue processing times was found only for the least discriminable cue CD1 and not for the other cues. This could happen due to unequal learning of cues of different discriminability levels after participants get more familiar with the cue. The individual response times depicted in Figure 6 and Figure 7 were recorded during the cue sensitivity test when participants were presented with the cue for the first time. We suggest that with practice participants become more familiar with the cues, leading to an overall shorter processing time of the cues. It is proposed that during the main experiment, the processing of the more difficult cue, CD1, remained temporally demanding, whereas, for the simpler and more discriminable cues, CD2 and CD3, participants required shorter processing times than in the cue sensitivity test. Therefore, the correlation between the individual cue processing times and performance at different interstimulus intervals was found only for the most complex and least discriminable cue but not for the more discriminable ones. In [23,33], the authors also did not find significant differences in the attentional effect for the simplest cues such as eye-gaze and arrows at various SOA. Furthermore, from the work in [23], no significant difference emerged for a more complex texture cue compared to the simpler ones, although a trend of a larger validity effect for a more complex cue was observed for larger SOA. The results of our study conform to previous findings showing no significant difference in attentional performance at different ISI for less complex cues. Nonetheless, a positive trend of correlation of performance at two different ISI with the individual cue processing time for the most complex cue indicates that the individual cue processing time can affect the temporal course of attentional effect for the most complex cue in the time range used in the present study. Testing more inter-stimulus intervals as well as different cue display times is of interest for further investigation of cueing effect on the temporal course of attention [39].

It is important to comment on the limitations of the present study. The results in Figure 5 suggest a gradual effect of cue discriminability on the attentional performance, however the difference for the slopes at the supra-threshold cue discriminability levels CD2 and CD3 was close to, but not, significant. This could be contributed by the ceiling effect caused by a challenging paradigm design with parametrically modulated endogenous cue discriminability. The difficulty of the task and individual variability, as well as a small sample size of this verification study, could also partially offset the low significance level. Furthermore, it is possible that the performance differences for the corresponding cue discriminability levels partly originate from incorrect decoding of the cue direction, particularly for the most complex cue. Specifically, to be sure about actual perceived cue direction in the main experiment, in each trial one would have to collect an additional response from participants. It would, however, further increase the load of the task for the participants and make it more tiring. This verification study intended to verify a gradual effect of cue complexity on attentional benefits and costs. Small sample size was, thus, tested for this preliminary feedback purpose. Nonetheless, the results of this verification study hint towards the scaling of attentional benefits and costs with cue discriminability which can be useful when a flexible allocation of attentional resources is facilitating performance. In future studies, a larger number of participants, as well as a bigger set of cue discriminability levels, could be used to get more insight into attentional scaling.

Generally, when discussing potential visual cue design with the purpose of improving a person’s performance, it is rather counterintuitive to develop a more complex or less discriminable cue. The rationale in this study, however, was to show that the cue properties can be tailored to vary the relation between attentional benefits and costs induced by the cue, but at the same time, one has to keep in mind that this tuning is limited by the emerging additional cue processing temporal costs. Considering the increasing tendency towards rather subtle visual cues in contrast to strong symbolic cues in diverse applications, in future validation studies, it would be interesting to test whether it is possible to find a “sweet spot” of cue complexity for attention. Namely, whether the cue can be discriminable just enough to bring the performance represented by attentional benefits and costs to its maximum, but does not impose significant cue processing costs.

To conclude, first, attentional performance seems to resemble a scaling behavior with varying properties of a spatial endogenous cue, specifically, its discriminability, even when cue discriminability is above the threshold level. Second, individual temporal differences in the processing of complex cues can impact attentional performance, particularly if the cue is complex. Thus, when designing endogenous cues and tuning their properties to purposely guide the user’s attention, it is important to consider the temporal processing costs of the cue, specifically, in case of a high cue complexity, but not for simple cues. The current verification study serves as an insight into the potential design of visual cues aiming to improve people’s performance. The main implication of this study is that to improve the total performance of the user over numerous locations using a flexible visual cue design, it is important to consider the trade-off between the cue complexity and its benefits vs. costs effect, as well as the individual processing time of the cue.

## Data Availability

Publicly available datasets were analyzed in this study. This data can be found here (accessed on 3 March 2021): https://osf.io/yfbqt/.

## Supplementary Materials

The following are available online at https://www.mdpi.com/article/10.3390/vision5020018/s1.

## Abbreviations

The following abbreviations are used in this manuscript:

2AFC	two-alternative-forced-choice
SOA	stimulus-onset asynchrony
ISI	inter-stimulus interval
