# Supplementary material for: The Impact of Shape-Based Cue Discriminability on Attentional Performance
Source: Vision (Basel). 2021 Apr 15;5(2):18. doi: 10.3390/vision5020018 (PMC8167570; doi:10.3390/vision5020018)
Supplement: Supplementary file 1 [file vision-05-00018-s001.zip › vision-1115026-supplementary.pdf]

Supplemetary information

S1 Generating the shape-based cue

As described in the main text in Section 2.2.2, the shape-based cue was an irregular radial frequency pattern with six lobes. The six lobes appeared to be enough to keep the possibility of smooth modulation of discriminability (compared to, for example, four lobes), and at the same time not visually overloading. In Figure S1 a representation of the cue is shown, where a cycle corresponding to each of the spatial frequencies is represented in a different color. To generate the set of cues for the experiment, the starting six spatial frequencies of the irregular spatial frequency pattern lobes were set to $\frac{42}{9}, \frac{42}{6}, \frac{42}{6}, \frac{42}{9}, \frac{42}{6}, \frac{42}{6}$, where the first value corresponds to the cueing lobe and the order continues counterclockwise along the pattern. Note, that the fractions are set such that the sum of all six denominators gives the common numerator, resulting in full span of polar angles from 0 to 360 degrees. Here it is an arbitrarily chosen number 42. As the starting point, the radial frequency of the two representative lobes (in Figure S1 B, pointing to the left and right, in color version: red and black) were set to a smaller value $\frac{42}{9}$ compared to the remaining four lobes (in Figure S1 B, pointing up and down, in color version: blue, green, turquoise and) which was set to $\frac{42}{6}$. As described in the main text Section 2.2.2, by modulating the ratio of the two representative lobes, a set of cues with varying cue discriminability was generated.

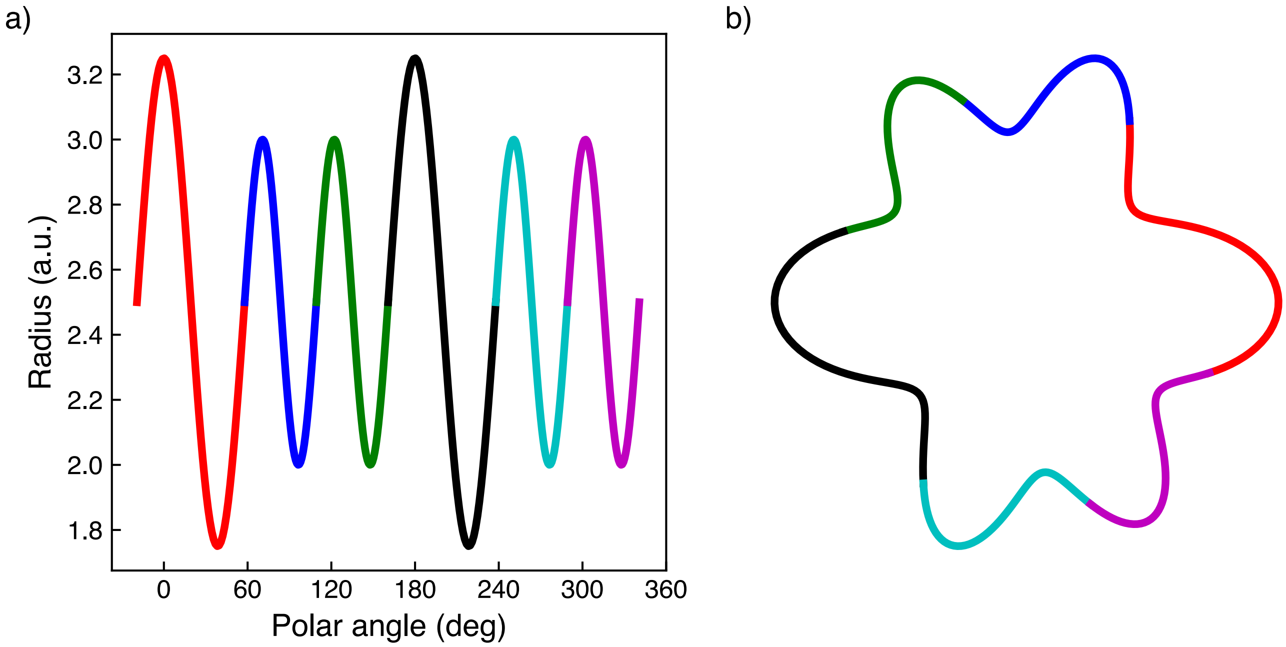

Figure S1: A representation of the shape-based cue. The different colors indicate each of the six cycles with respective radial frequencies. a) The radius R as a function of the polar angle θ. The mean radius R_0 was set to 2.5 a.u.; b) Radial frequency pattern in polar coordinates. For the notations please refer to the main text Equation 1.

S2 Unnormalized group data

In Figure S2 the unnormalized group data for 8 measured subjects is shown. Specifically, the unnormalized slope in the invalid and valid conditions for each cue discriminability level, as well as the slope in the neutral condition, is presented.

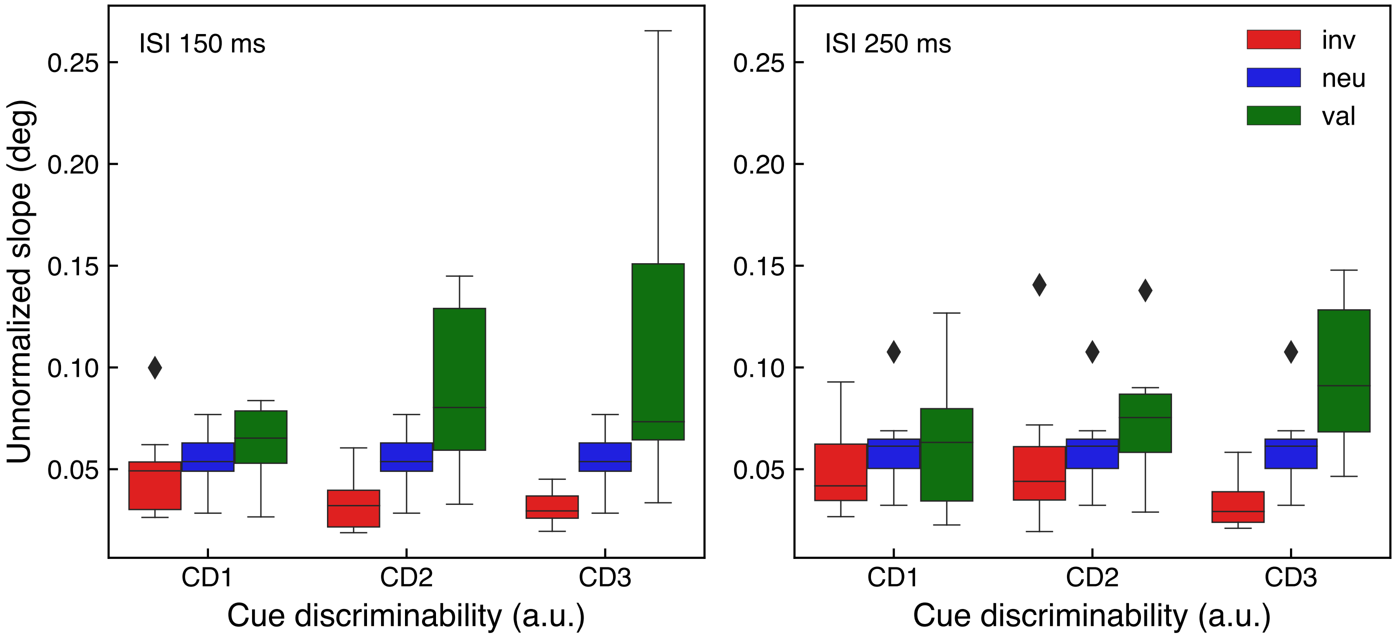

Figure S2: The group data for 8 measured subjects of the unnormalized slopes in the invalid (red), neutral (blue) and valid (green) conditions. Note, that the slope in the neutral condition is independent from the cue discriminability level and is, thus, constant for the respective inter-stimulus intervals.

S3 Deviance

In Figure S3 the distribution of the deviance of all fits is shown. As discussed in the main text, the deviance varies across the validity which is expected as the invalid condition is particularly challenging. The invalid condition also has less measured data points than valid or neutral conditions, to keep the cue informative, which results in higher noise levels for the invalid condition. Nonetheless, for the purpose of the pilot study described in the main text, the trend of slopes across validity and cue discriminability extracted from presented data was evaluated.

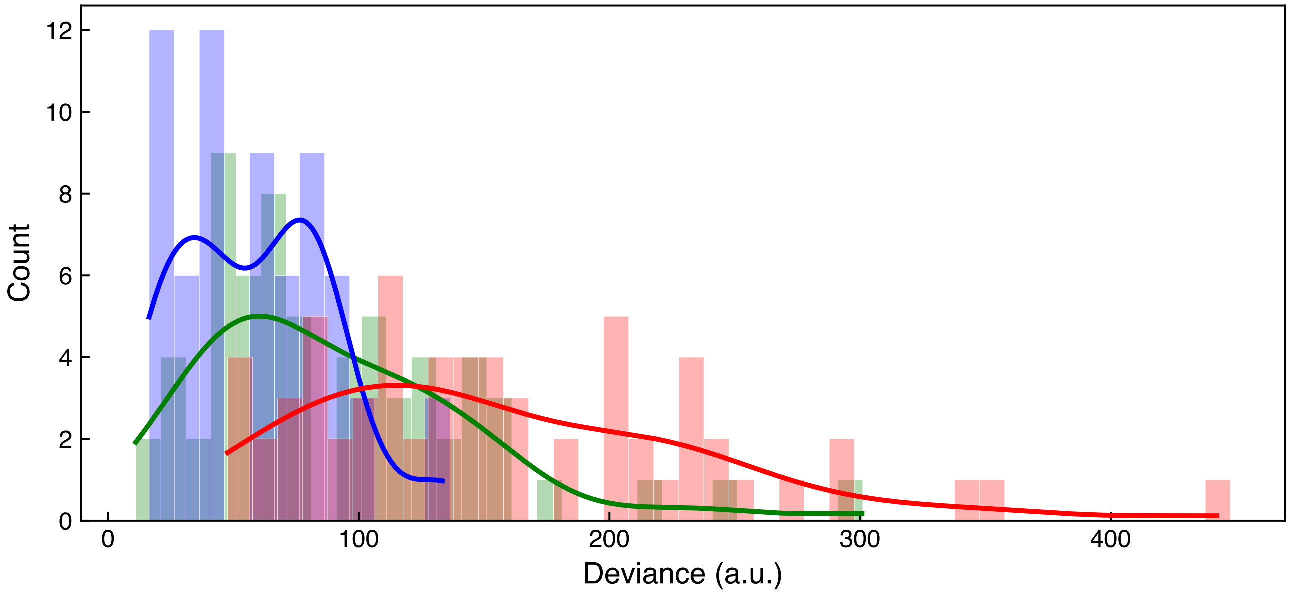

Figure S3: Deviance distributions for valid (green), neutral (blue) and invalid (red) validity conditions. The bin size is set to 10. The solid lines represent the kernel density estimate.

S4 Individual data

In Figure S4 – Figure S25 the fits of psychometric functions for all 11 measure subjects are shown at two ISI, respectively. In Figure S10 and Figure S11 the data for one of the three excluded subjects (CueD_11) is presented, who explicitly reported that he ignored the cue. In Figure S22 – Figure S25 the data for the remaining two subjects (CueD_17 and CueD_18) is shown, for whom the noise level was consistently high across all experimental conditions.

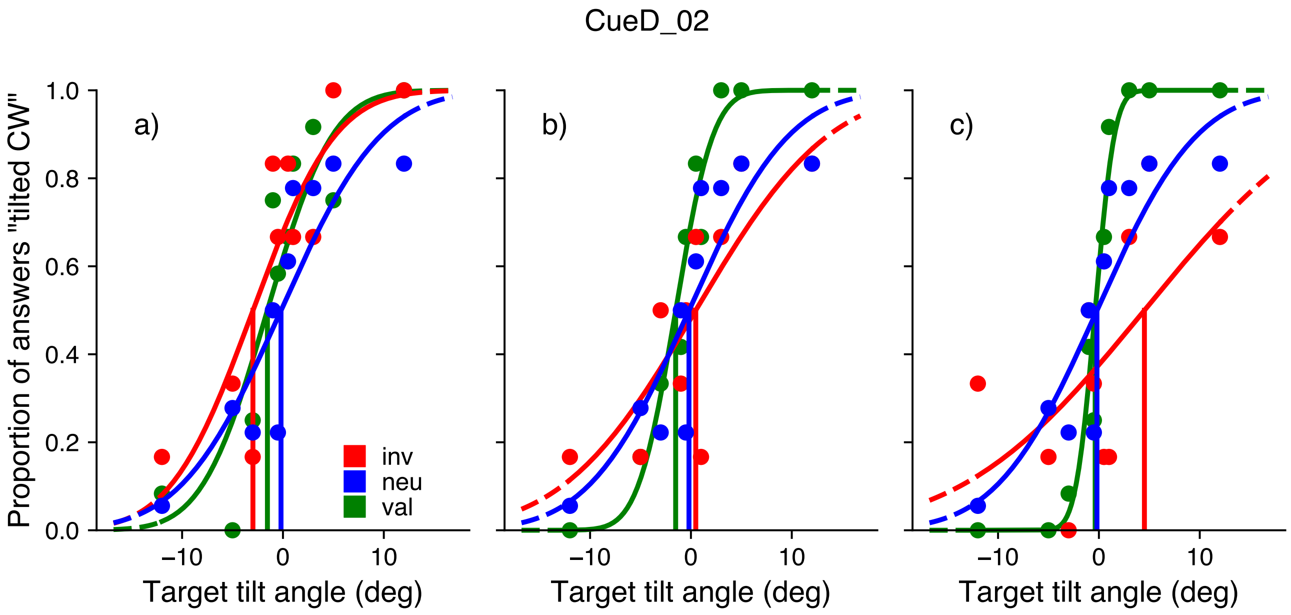

Figure S4: A family of psychometric fits obtained for subject CueD_02 at ISI 150 ms. Green, blue and red curves correspond to the valid, neutral and invalid conditions, respectively. The data sets correspond to cue discriminability levels a) CD1, b) CD2, and c) CD3. The title of the figure is the pseudonym of the respective subject.

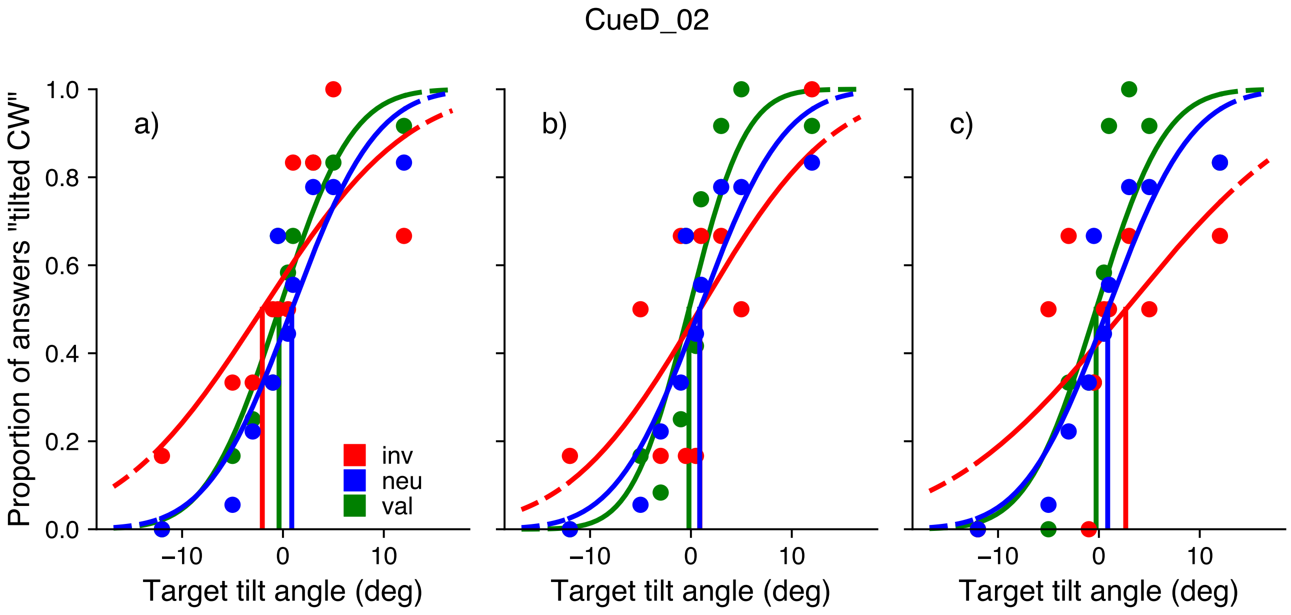

Figure S5: A family of psychometric fits obtained for subject CueD_02 at ISI 250 ms. Green, blue and red curves correspond to the valid, neutral and invalid conditions, respectively. The data sets correspond to cue discriminability levels a) CD1, b) CD2, and c) CD3. The title of the figure is the pseudonym of the respective subject.

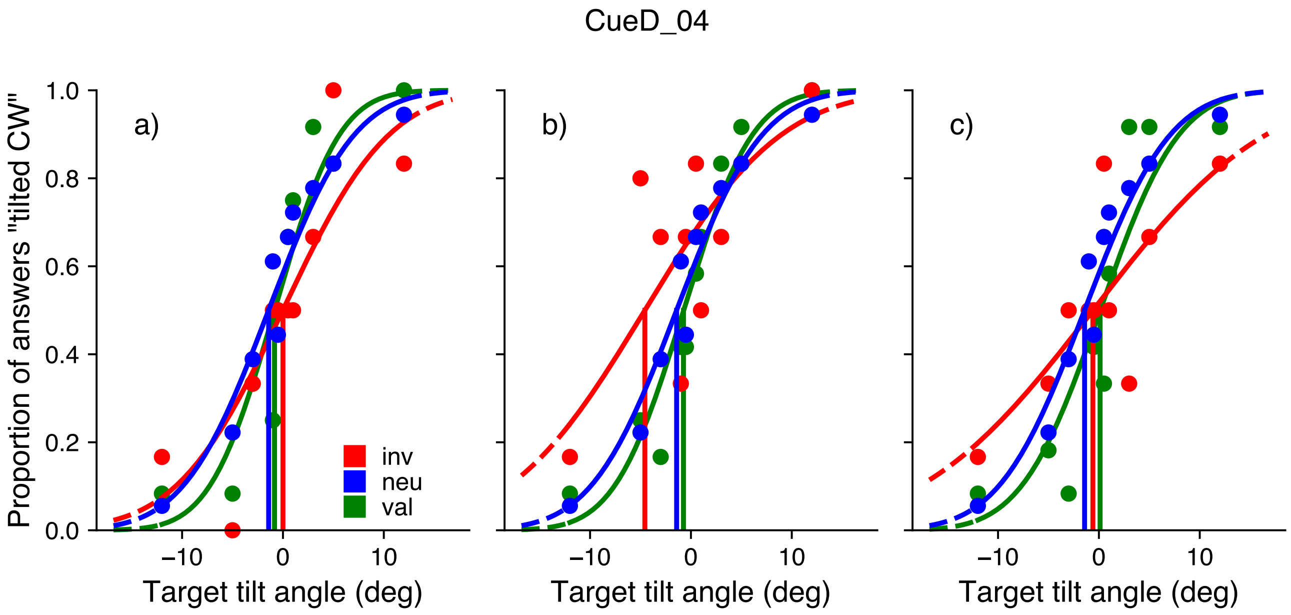

Figure S6: A family of psychometric fits obtained for subject CueD_04 at ISI 150 ms. Green, blue and red curves correspond to the valid, neutral and invalid conditions, respectively. The data sets correspond to cue discriminability levels a) CD1, b) CD2, and c) CD3. The title of the figure is the pseudonym of the respective subject.

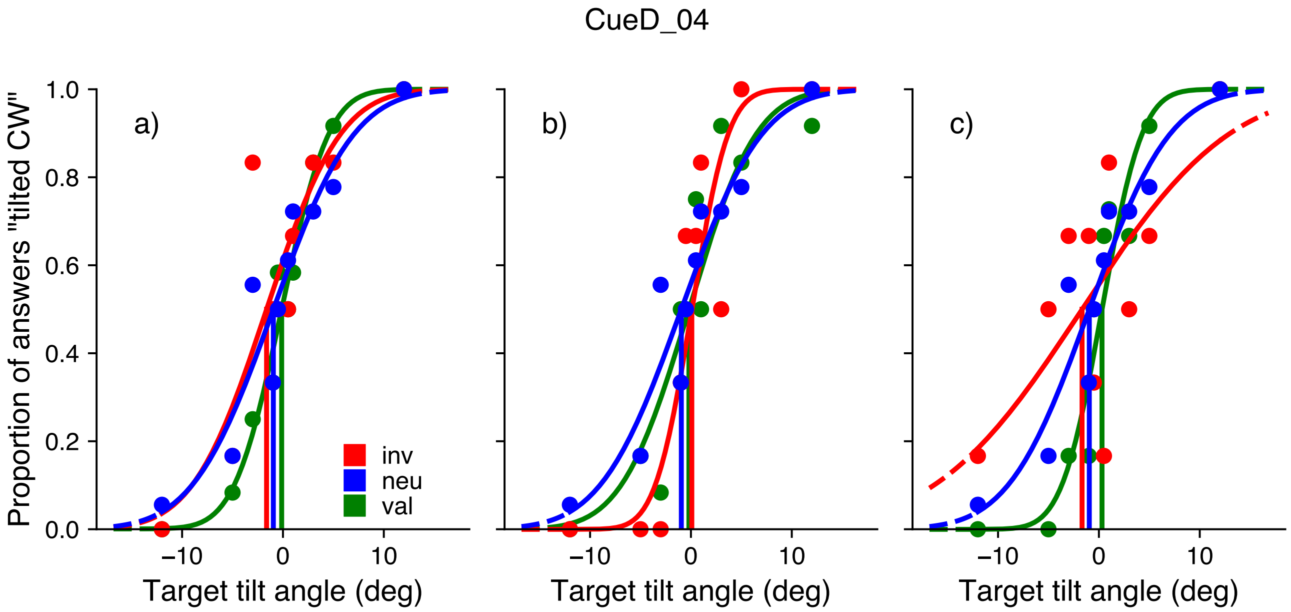

Figure S7: A family of psychometric fits obtained for subject CueD_04 at ISI 250 ms. Green, blue and red curves correspond to the valid, neutral and invalid conditions, respectively. The data sets correspond to cue discriminability levels a) CD1, b) CD2, and c) CD3. The title of the figure is the pseudonym of the respective subject.

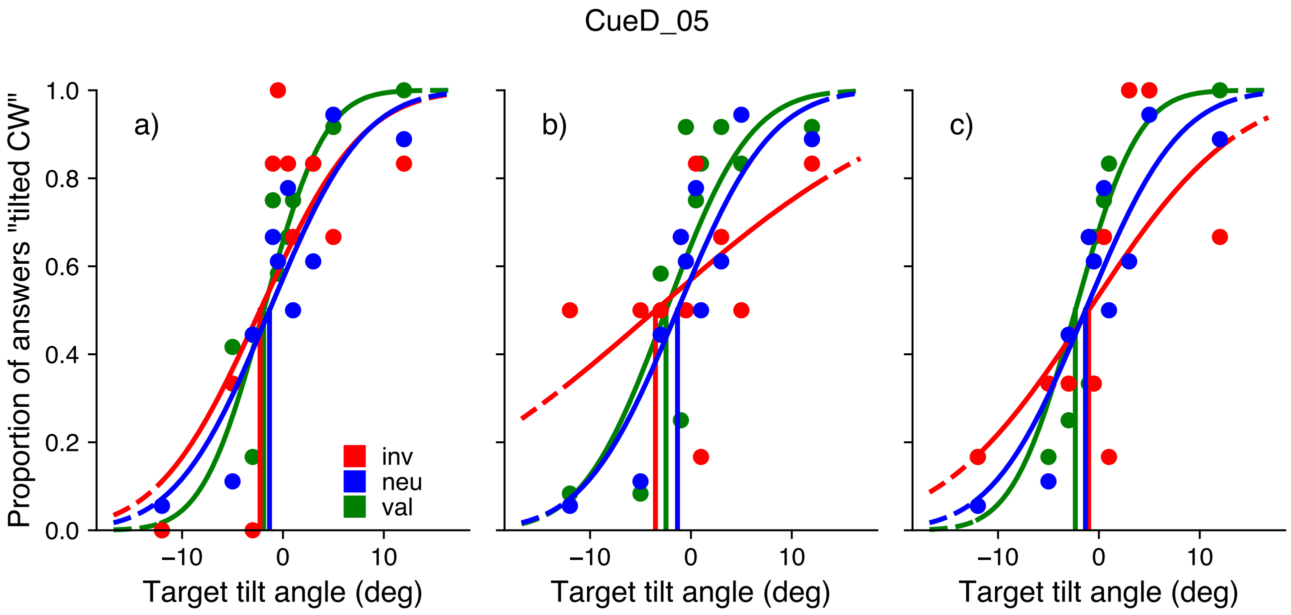

Figure S8: A family of psychometric fits obtained for subject CueD_05 at ISI 150 ms. Green, blue and red curves correspond to the valid, neutral and invalid conditions, respectively. The data sets correspond to cue discriminability levels a) CD1, b) CD2, and c) CD3. The title of the figure is the pseudonym of the respective subject.

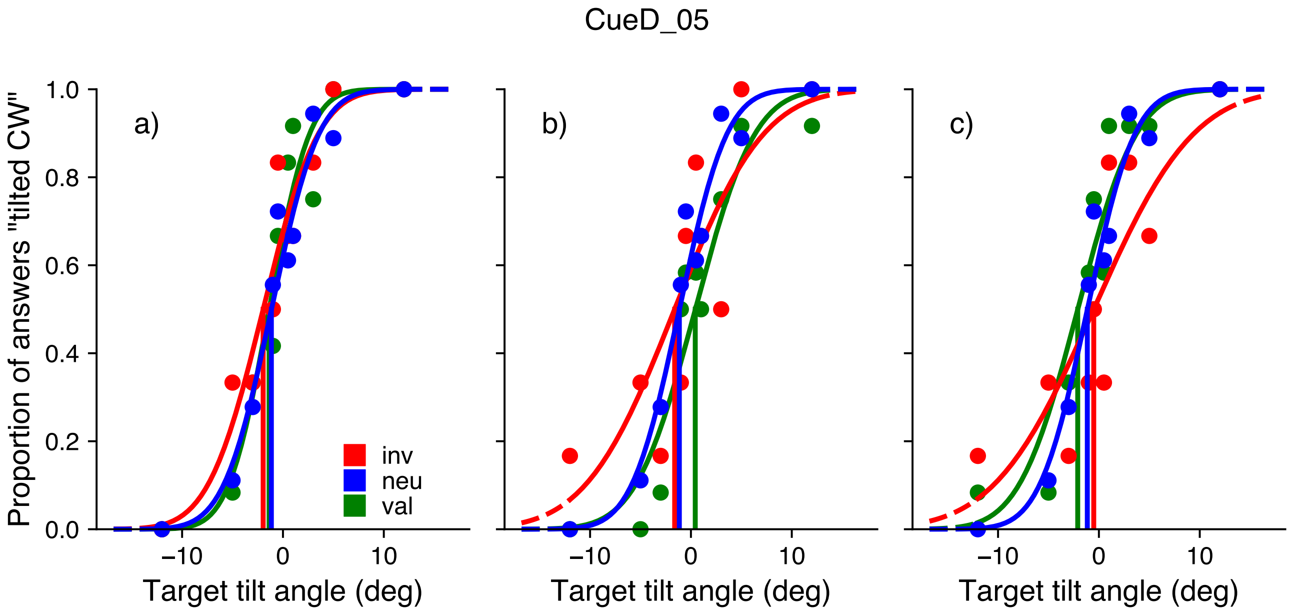

Figure S9: A family of psychometric fits obtained for subject CueD_05 at ISI 250 ms. Green, blue and red curves correspond to the valid, neutral and invalid conditions, respectively. The data sets correspond to cue discriminability levels a) CD1, b) CD2, and c) CD3. The title of the figure is the pseudonym of the respective subject.

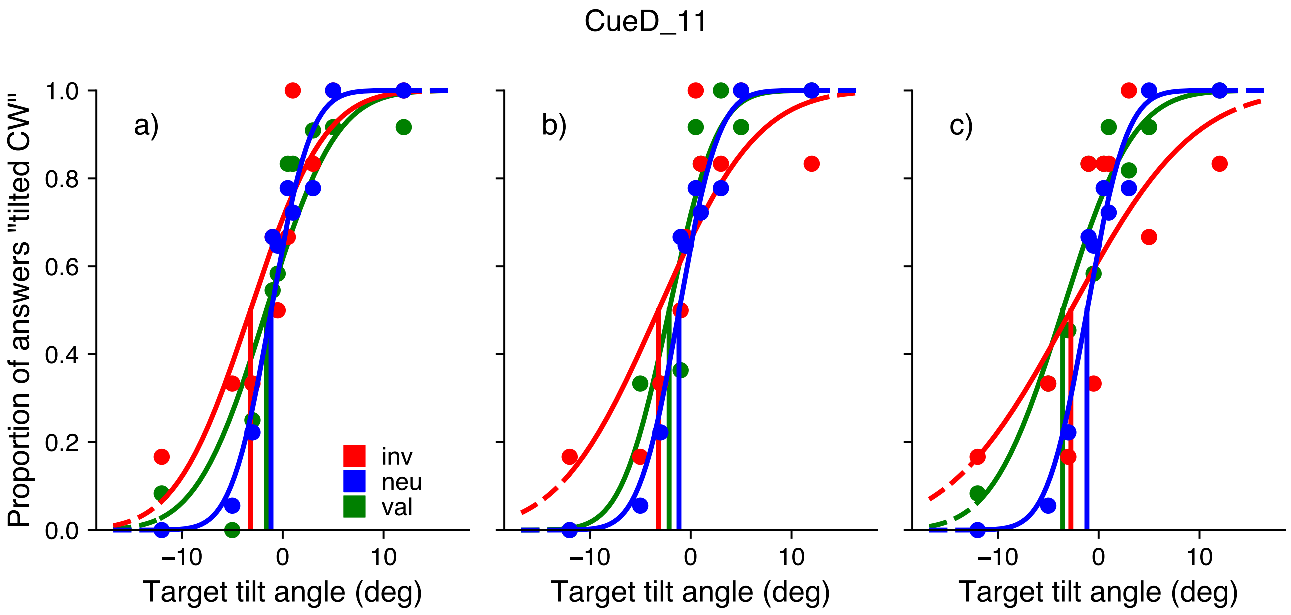

Figure S10: A family of psychometric fits obtained for subject CueD_11 at ISI $150\,\mathrm{ms}$. Green, blue and red curves correspond to the valid, neutral and invalid conditions, respectively. The data sets correspond to cue discriminability levels a) CD1, b) CD2, and c) CD3. The title of the figure is the pseudonym of the respective subject.

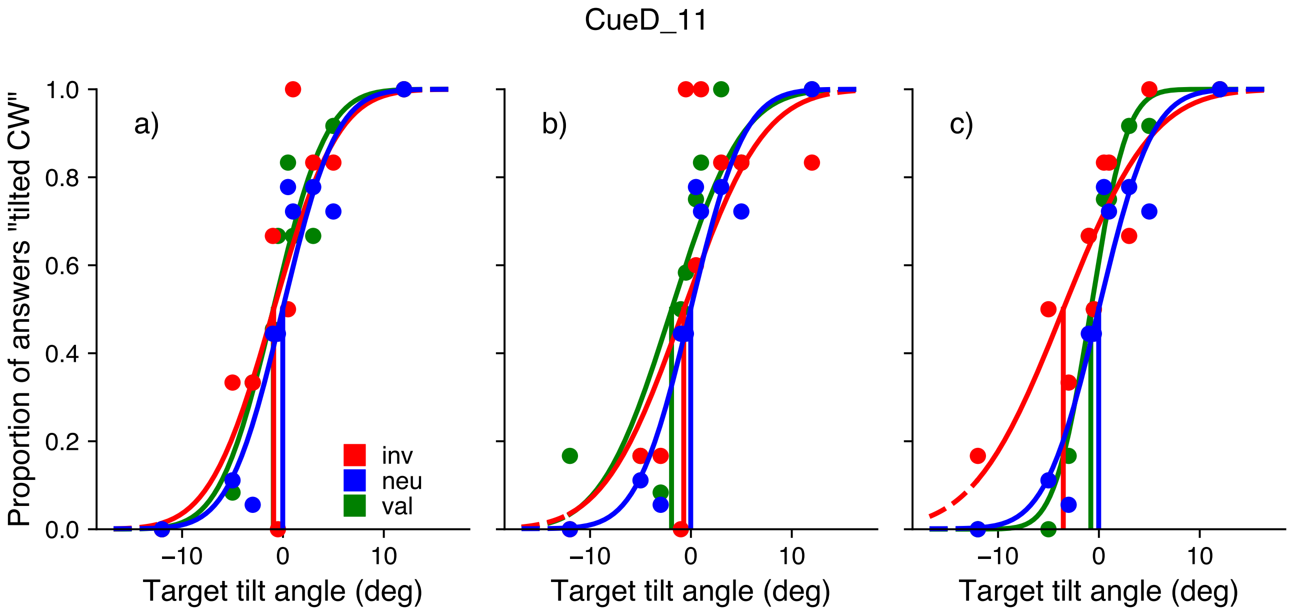

Figure S11: A family of psychometric fits obtained for subject CueD_11 at ISI $250\,\mathrm{ms}$. Green, blue and red curves correspond to the valid, neutral and invalid conditions, respectively. The data sets correspond to cue discriminability levels a) CD1, b) CD2, and c) CD3. The title of the figure is the pseudonym of the respective subject.

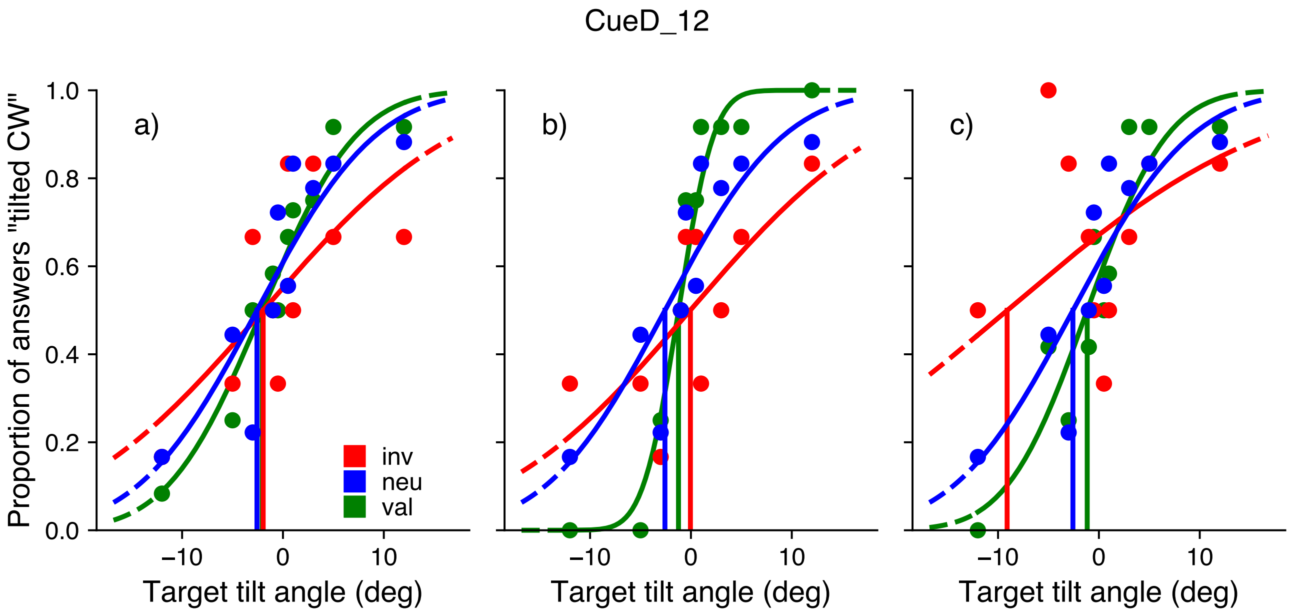

Figure S12: A family of psychometric fits obtained for subject CueD_12 at ISI $150\,\text{ms}$. Green, blue and red curves correspond to the valid, neutral and invalid conditions, respectively. The data sets correspond to cue discriminability levels a) CD1, b) CD2, and c) CD3. The title of the figure is the pseudonym of the respective subject.

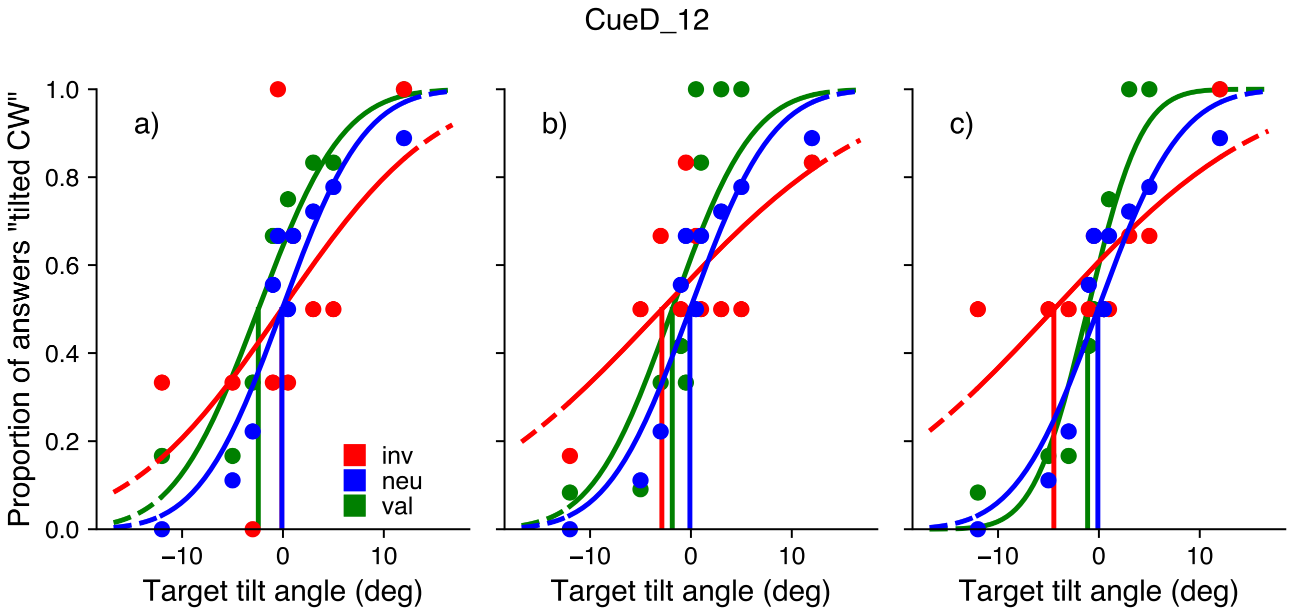

Figure S13: A family of psychometric fits obtained for subject CueD_12 at ISI $250\,\text{ms}$. Green, blue and red curves correspond to the valid, neutral and invalid conditions, respectively. The data sets correspond to cue discriminability levels a) CD1, b) CD2, and c) CD3. The title of the figure is the pseudonym of the respective subject.

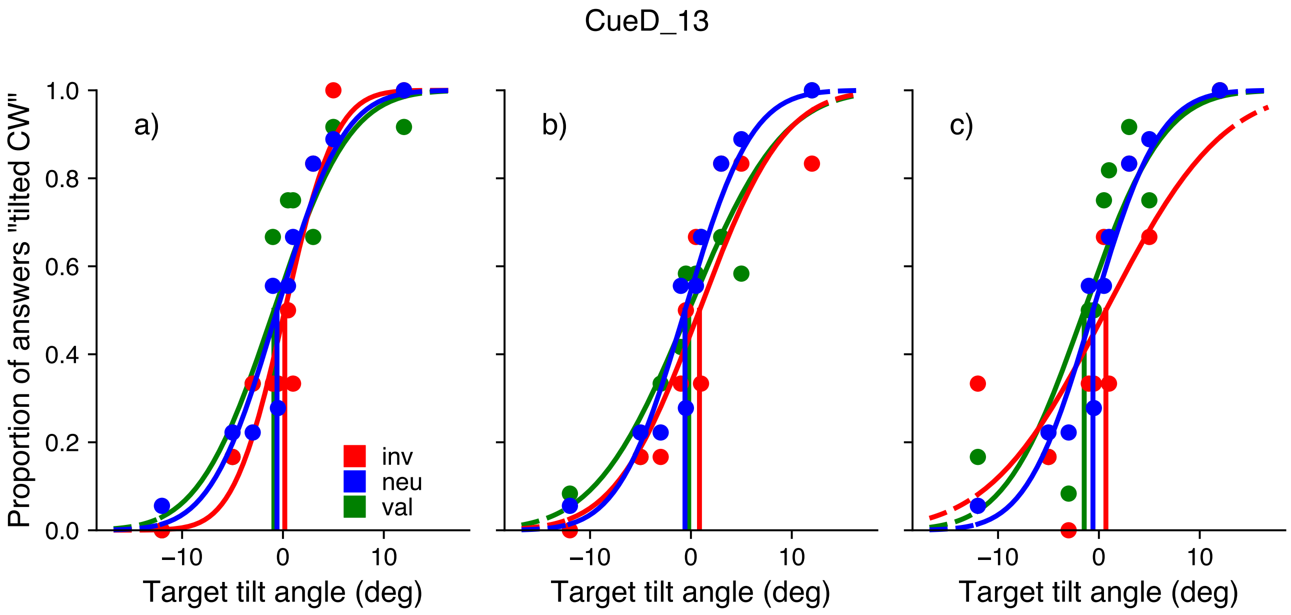

Figure S14: A family of psychometric fits obtained for subject CueD_13 at ISI $150\,\mathrm{ms}$. Green, blue and red curves correspond to the valid, neutral and invalid conditions, respectively. The data sets correspond to cue discriminability levels a) CD1, b) CD2, and c) CD3. The title of the figure is the pseudonym of the respective subject.

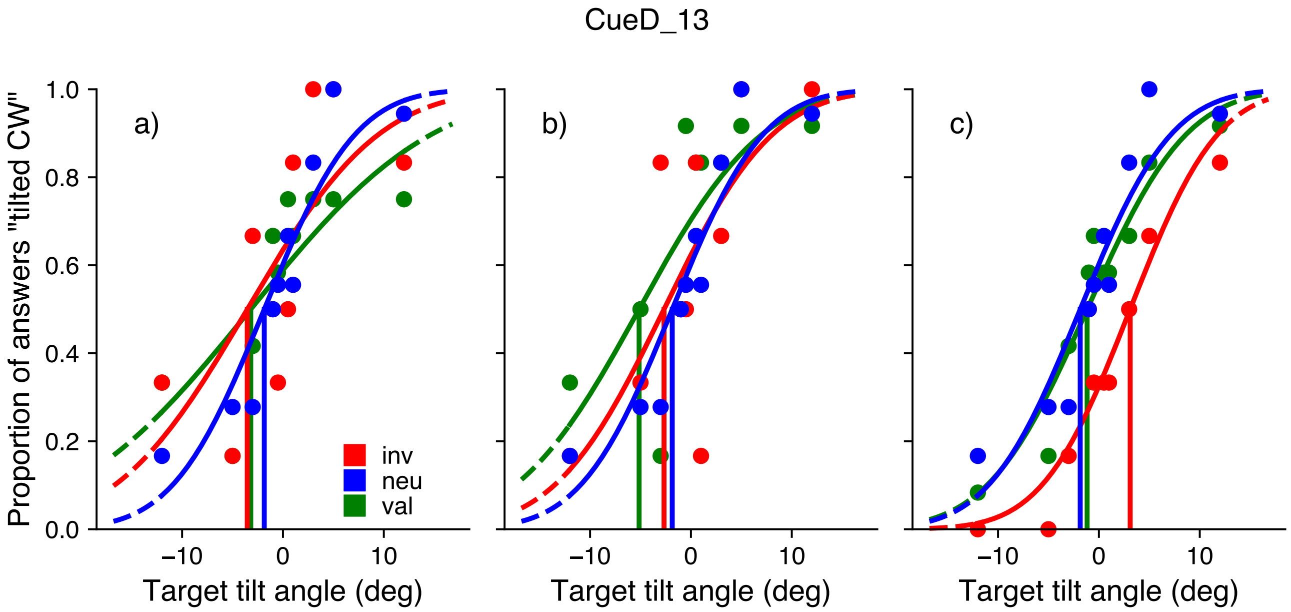

Figure S15: A family of psychometric fits obtained for subject CueD_13 at ISI $250\,\mathrm{ms}$. Green, blue and red curves correspond to the valid, neutral and invalid conditions, respectively. The data sets correspond to cue discriminability levels a) CD1, b) CD2, and c) CD3. The title of the figure is the pseudonym of the respective subject.

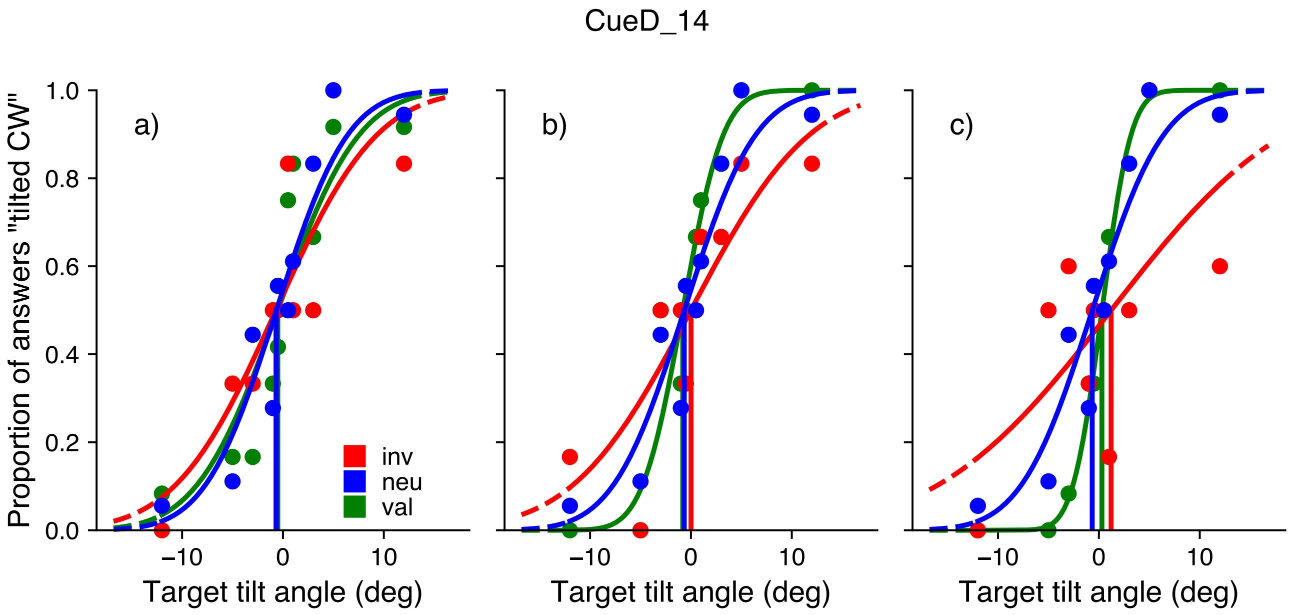

Figure S16: A family of psychometric fits obtained for subject CueD_14 at ISI 150 ms. Green, blue and red curves correspond to the valid, neutral and invalid conditions, respectively. The data sets correspond to cue discriminability levels a) CD1, b) CD2, and c) CD3. The title of the figure is the pseudonym of the respective subject.

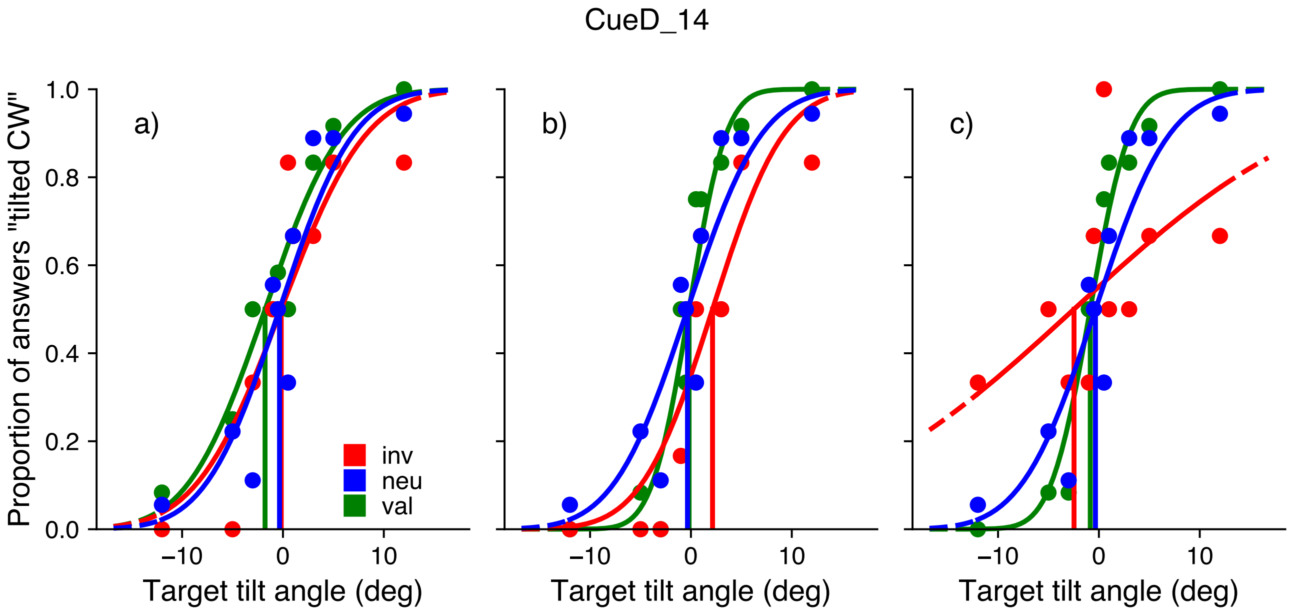

Figure S17: A family of psychometric fits obtained for subject CueD_14 at ISI 250 ms. Green, blue and red curves correspond to the valid, neutral and invalid conditions, respectively. The data sets correspond to cue discriminability levels a) CD1, b) CD2, and c) CD3. The title of the figure is the pseudonym of the respective subject.

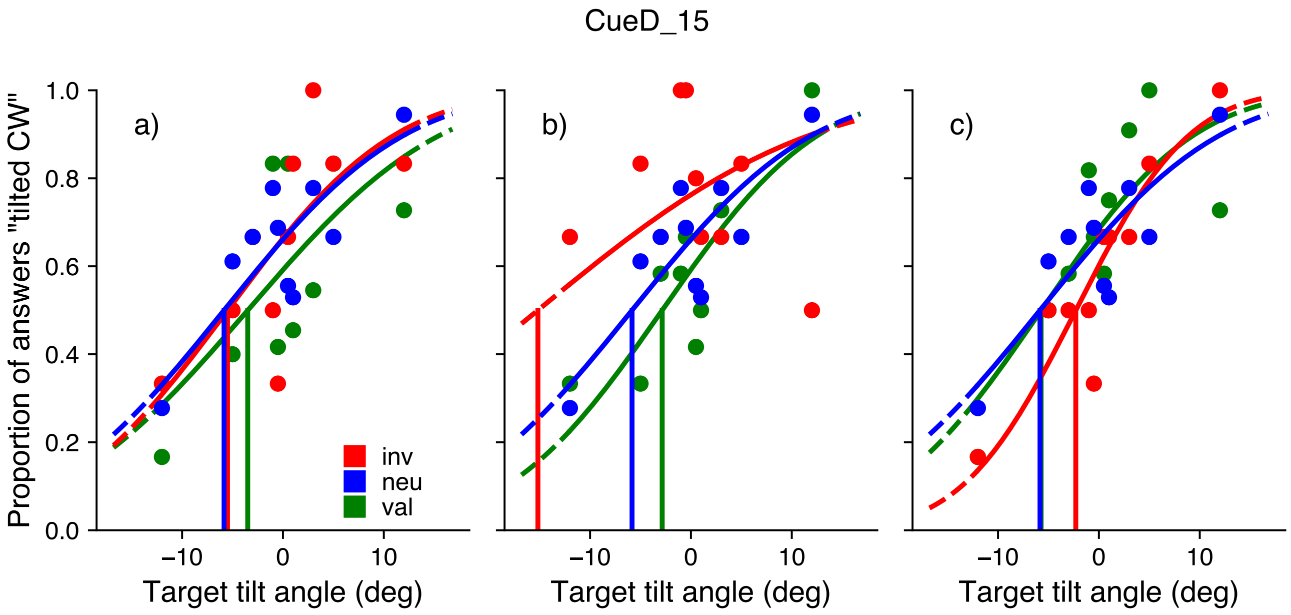

Figure S18: A family of psychometric fits obtained for subject CueD_15 at ISI $150\,\mathrm{ms}$. Green, blue and red curves correspond to the valid, neutral and invalid conditions, respectively. The data sets correspond to cue discriminability levels a) CD1, b) CD2, and c) CD3. The title of the figure is the pseudonym of the respective subject.

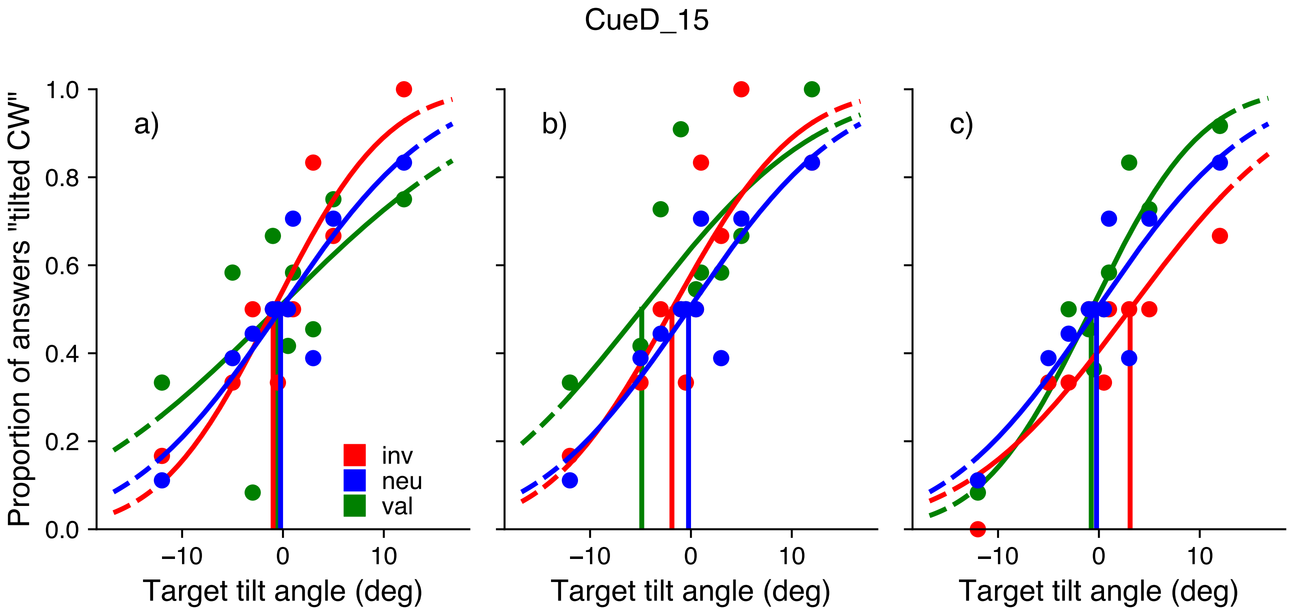

Figure S19: A family of psychometric fits obtained for subject CueD_15 at ISI $250\,\mathrm{ms}$. Green, blue and red curves correspond to the valid, neutral and invalid conditions, respectively. The data sets correspond to cue discriminability levels a) CD1, b) CD2, and c) CD3. The title of the figure is the pseudonym of the respective subject.

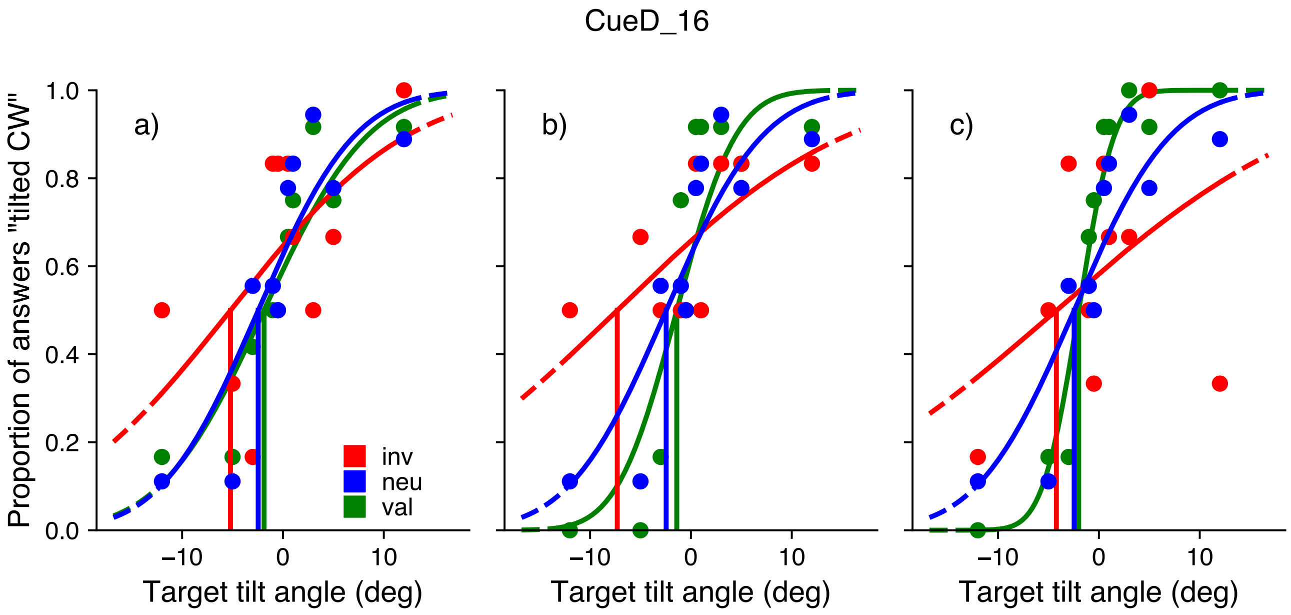

Figure S20: A family of psychometric fits obtained for subject CueD_16 at ISI 150 ms. Green, blue and red curves correspond to the valid, neutral and invalid conditions, respectively. The data sets correspond to cue discriminability levels a) CD1, b) CD2, and c) CD3. The title of the figure is the pseudonym of the respective subject.

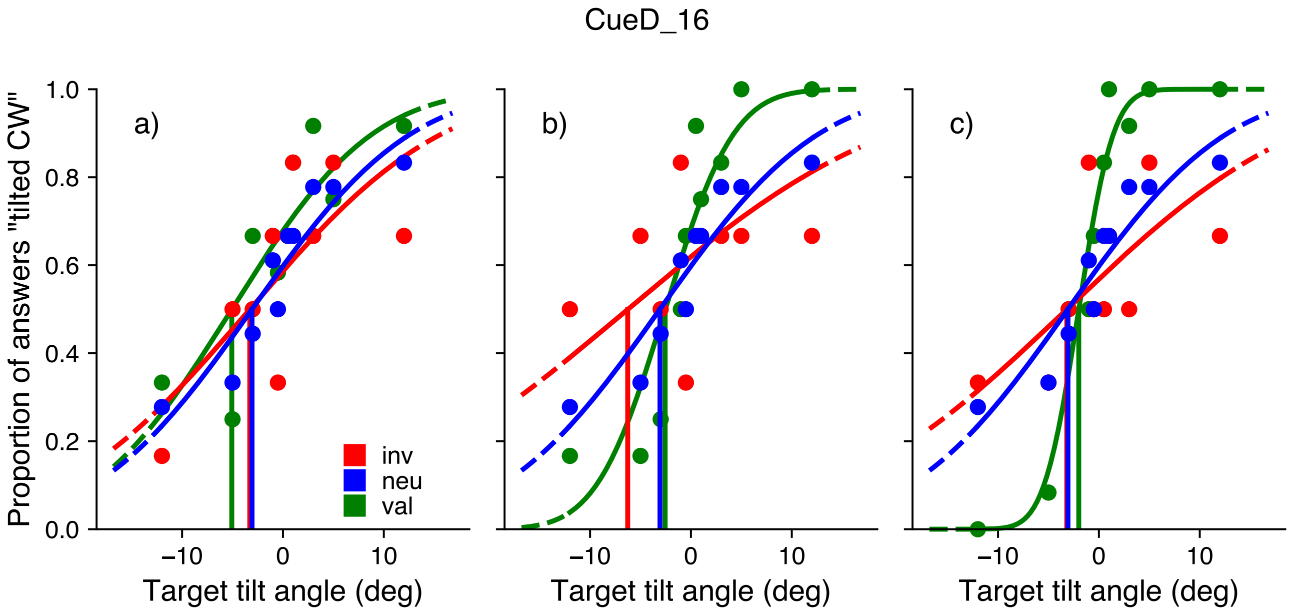

Figure S21: A family of psychometric fits obtained for subject CueD_16 at ISI 250 ms. Green, blue and red curves correspond to the valid, neutral and invalid conditions, respectively. The data sets correspond to cue discriminability levels a) CD1, b) CD2, and c) CD3. The title of the figure is the pseudonym of the respective subject.

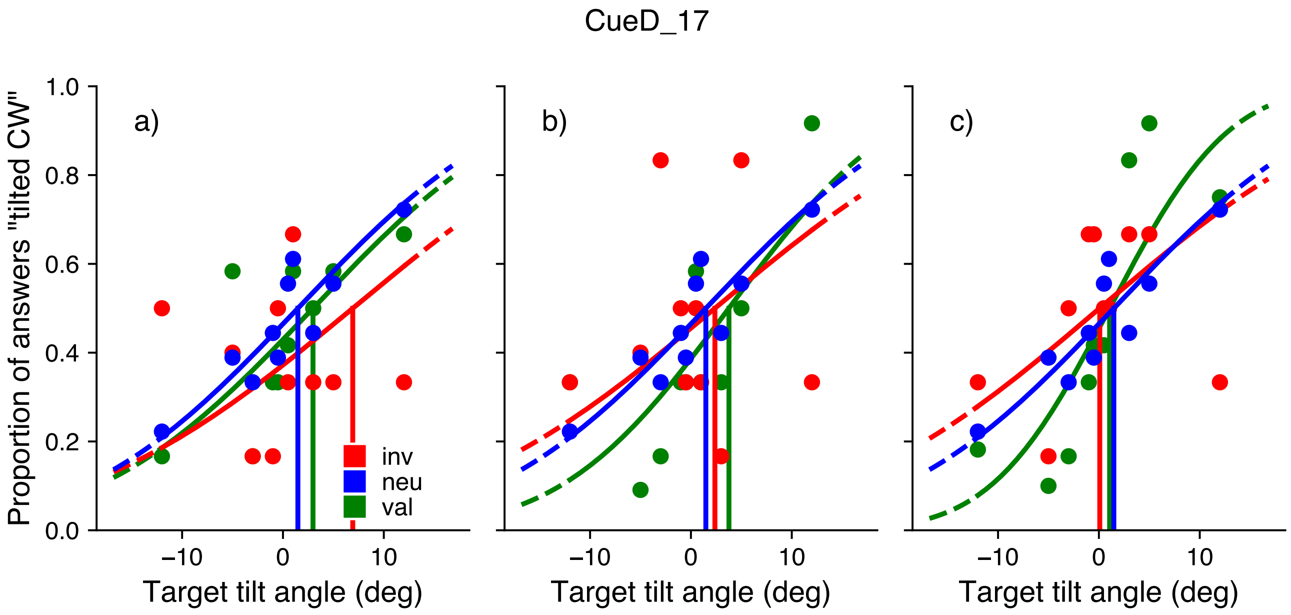

Figure S22: A family of psychometric fits obtained for subject CueD_17 at ISI 150 ms. Green, blue and red curves correspond to the valid, neutral and invalid conditions, respectively. The data sets correspond to cue discriminability levels a) CD1, b) CD2, and c) CD3. The title of the figure is the pseudonym of the respective subject.

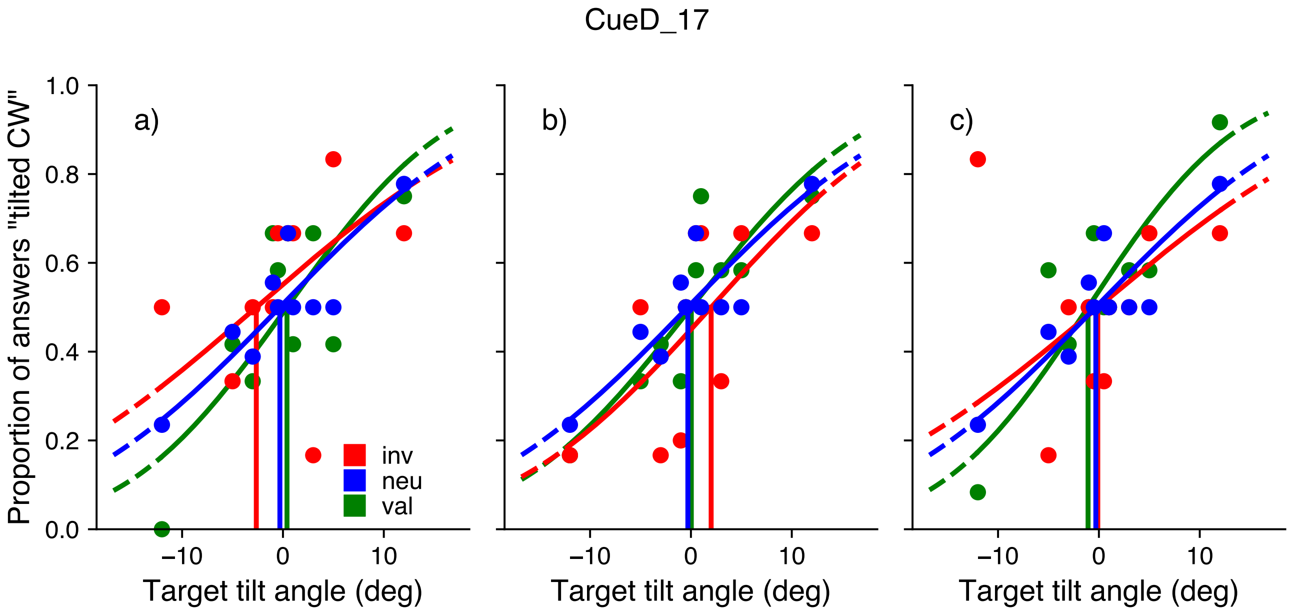

Figure S23: A family of psychometric fits obtained for subject CueD_17 at ISI 250 ms. Green, blue and red curves correspond to the valid, neutral and invalid conditions, respectively. The data sets correspond to cue discriminability levels a) CD1, b) CD2, and c) CD3. The title of the figure is the pseudonym of the respective subject.

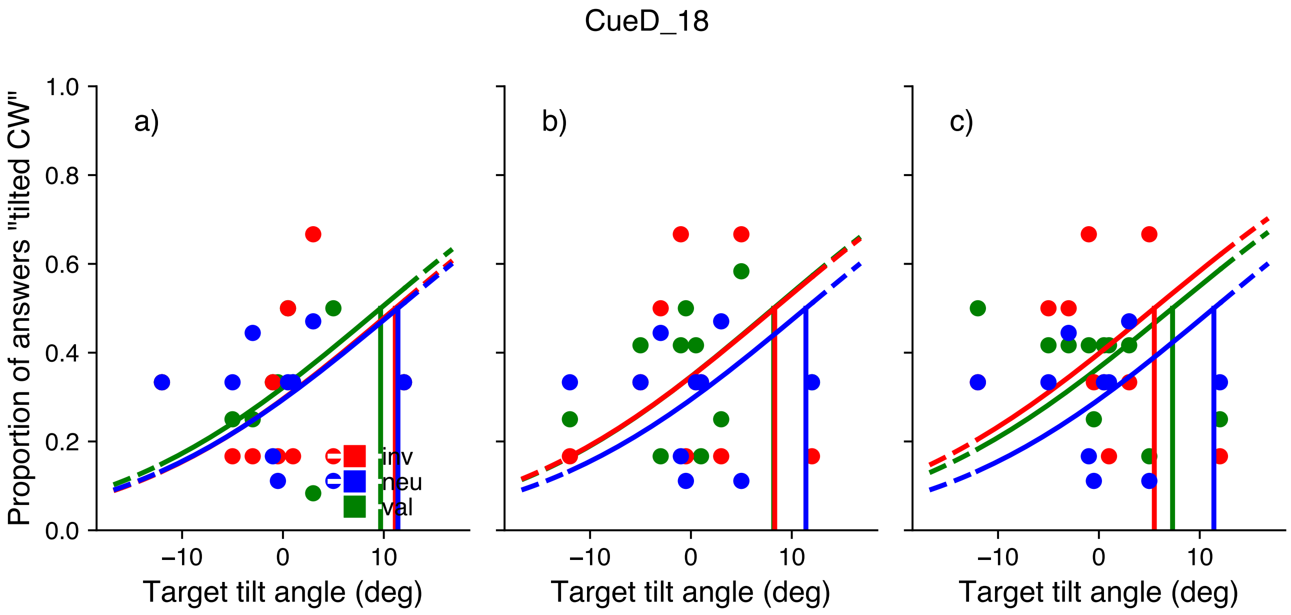

Figure S24: A family of psychometric fits obtained for subject CueD_18 at ISI $150\,\mathrm{ms}$. Green, blue and red curves correspond to the valid, neutral and invalid conditions, respectively. The data sets correspond to cue discriminability levels a) CD1, b) CD2, and c) CD3. The title of the figure is the pseudonym of the respective subject.

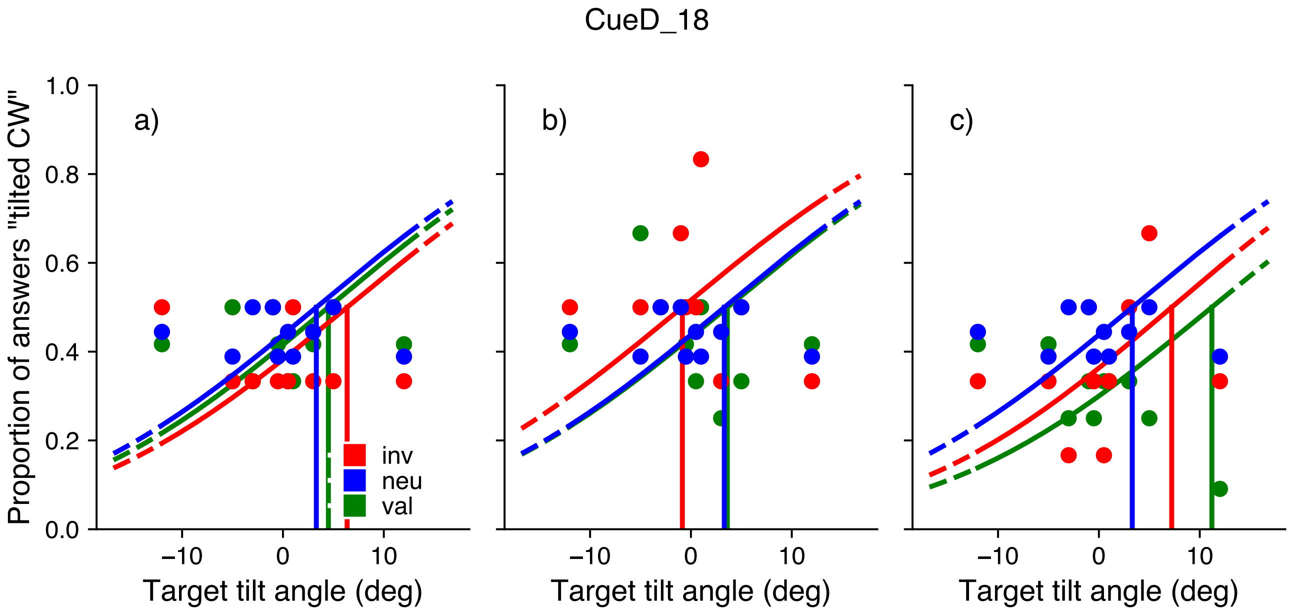

Figure S25: A family of psychometric fits obtained for subject CueD_18 at ISI $250\,\mathrm{ms}$. Green, blue and red curves correspond to the valid, neutral and invalid conditions, respectively. The data sets correspond to cue discriminability levels a) CD1, b) CD2, and c) CD3. The title of the figure is the pseudonym of the respective subject.
